# Combination of abiotic stresses accelerates photoprotection in exceptionally chilling-tolerant C_4_ grass by fine-tuning the content of multiple pigments

**DOI:** 10.1093/jxb/erag236

**Published:** 2026-05-21

**Authors:** Benjamin Turc, Asha Kumari, Maria Carter, Katarzyna Glowacka

**Affiliations:** Department of Biochemistry and Center for Plant Science Innovation, University of Nebraska-Lincoln, Lincoln, NE 68588, USA; Department of Biochemistry and Center for Plant Science Innovation, University of Nebraska-Lincoln, Lincoln, NE 68588, USA; Department of Biochemistry and Center for Plant Science Innovation, University of Nebraska-Lincoln, Lincoln, NE 68588, USA; Department of Biochemistry and Center for Plant Science Innovation, University of Nebraska-Lincoln, Lincoln, NE 68588, USA; Polish Academy of Sciences, Institute of Plant Genetics, Poznań 60-479, Poland; University of Essex, UK

**Keywords:** Abiotic stresses, anthocyanins, C_4_ photosynthesis, carotenoids, chlorophylls, hyperspectral indexes, *Miscanthus* sp, non-photochemical quenching, xanthophylls

## Abstract

C_4_ grasses are crucial for food and biofuel production. Originating from warm regions of the world, C_4_-photosynthesizing plants typically exhibit poor chilling tolerance. Some C_4_ grasses of *Miscanthus* are recognized for their exceptional chilling tolerance; however, the mechanism behind it is not fully understood. Here, we hypothesize that the rapid adjustment of leaf pigment composition contributes to mechanisms that protect photosynthesis during the initial short-term response to chilling. *Miscanthus* accessions with documented contrasting levels of chilling tolerance were subjected to chilling under dark and light conditions with or without nutrient limitations. The changes in pigment composition were assessed by hyperspectral indexes and molecularly validated. Our results showed that a high-chilling-tolerant accession accumulates zeaxanthin and anthocyanins while reducing chlorophyll content at the end of a night of chilling treatment when grown on low-fertility soil. Interestingly, at the end of the night, low-soil fertility alone was able to induce a significant difference in zeaxanthin accumulation between accessions. Zeaxanthin accumulated during the night led to 38% faster non-photochemical quenching on the following morning. Transcriptional differential regulation of enzymes involved in pigment anabolism and catabolism supports the dynamic adjustment in leaf pigment composition. The investigated changes in pigment composition can inspire new strategies to engineer crops for better stress resistance.

## Teaser

A *Miscanthus* high-chilling-tolerant accession accumulates zeaxanthin and anthocyanins while reducing chlorophyll content at the end of a night of chilling treatment in a stress-dependent fashion.

## Introduction

All life on earth depends on autotrophic organisms, such as plants, which ensure primary production in ecosystems by turning inorganic compounds into organic matter. Photosynthesis is performed by plants to achieve this primary production, using light energy and CO_2_ to produce biomass. While plants evolved different types of photosynthesis, the C_4_ type was shown to be more efficient than C_3_ in terms of light, nitrogen, and water use (Sage and Pearcy, 1987; Long and Spence, 2013; Way *et al.*, 2014; Wasilewska-Debowska *et al.*, 2022). As a result, C_4_ plants are among the most productive plants, accounting for 21% of global terrestrial carbon fixation despite representing only ∼3% of plant species (Lloyd and Farquhar, 1994). Furthermore, C_4_ warm-season grasses such as maize, sorghum, and sugarcane support 35% of the world’s cereal production (FAO *et al.*, 2023) while others are an important feedstock for biofuels (Langholtz *et al.*, 2016).

C_4_ photosynthesis originated from the tropics and subtropics, therefore, the C_4_ warm-season grasses largely show a poor tolerance to chilling, defined as a temperature between 4 °C and 14 °C, limiting their geographical distribution, establishment, and productivity under a temperate climate (Ma *et al.*, 2015; Langholtz *et al.*, 2016; Chiluwal *et al.*, 2018). Chilling impacts the activity of enzymes by reducing their reaction rates. As a consequence, less energy coming from the light can be used to assimilate CO_2_, leading to an excessive formation of reactive oxygen species (ROS; Y. Li *et al.*, 2020). Photosystems damaged by ROS are no longer efficient in providing energy to assimilate CO_2_ (Nouri *et al.*, 2015; Singh and Thakur, 2018; Sharma *et al.*, 2019; Muhammad *et al.*, 2021), which can stop plant growth and eventually lead to plant death (Allen and Ort, 2001; Głowacka *et al.*, 2014; Gómez-Muñoz *et al.*, 2018).

To prevent ROS accumulation during photosynthesis, plants have evolved a mechanism known as non-photochemical quenching (NPQ) of chlorophyll fluorescence, which primarily depends on the xanthophyll cycle involving violaxanthin and zeaxanthin (Müller *et al.*, 2001). Typically, under light conditions, violaxanthin is dynamically converted to zeaxanthin, and it has been suggested that zeaxanthin allosterically affects the interactions of PSII subunit S (PsbS) with minor antenna proteins, which are needed to fully rearrange PSII–light harvesting complexes II supercomplexes and consequent occurrence of NPQ (Betterle *et al.*, 2009; Correa-Galvis *et al.*, 2016; Sacharz *et al.*, 2017). Two of the fastest and most interconnected components of NPQ are energy-dependent quenching (qE) and zeaxanthin-dependent quenching (qZ) (Sacharz *et al.*, 2017; Steen *et al.*, 2020). NPQ dynamically responds to the light regime (Serôdio and Lavaud, 2011; Turc *et al.*, 2024). In the light, NPQ kinetics can be divided into attributes such as NPQ induction rate and maximum NPQ that are shaped by both qE and qZ (Glowacka, 2025). In the dark, which follows light exposure, the kinetics of NPQ can be divided into the relaxation rate and minimum NPQ. Fast relaxing qE mainly contributes to the rate of NPQ relaxation, and qZ mostly contributes to a residual NPQ that is NPQ not relaxed after a short dark incubation. The slow recovery from NPQ under low light conditions can cause a substantial reduction in photosynthetic efficiency, leading to yield loss (Kromdijk *et al.*, 2016; Cowling *et al.*, 2022). On the other hand, faster induction of NPQ might be required for swift de-excitation of excess energy under environmental conditions that limit photochemical quenching, including chilling and drought stresses (Jia *et al.*, 2020; Haupt and Glowacka, 2024). Anthocyanins are the other pigments that can play a role in photoprotection by acting as antioxidants, ROS scavengers, and light-shielding agents compensating for insufficient NPQ (Tanaka *et al.*, 2008; Zhu *et al.*, 2018; Yu *et al.*, 2023). Plants also tightly regulate chlorophyll metabolism, balancing chlorophyll biosynthesis and degradation, to precisely adjust the photosynthetic capacity to various developmental and environmental cues (Jarvis and Lopez-Juez, 2013; Wang *et al.*, 2020; Hu *et al.*, 2023; Sahay *et al.*, 2024a).

*Miscanthus*, a perennial, rhizomatous fast-growing C_4_ grass, is an important candidate for biomass production for biofuel and bioproducts (Somerville *et al.*, 2010; Li *et al.*, 2018; Clifton-Brown *et al.*, 2019). The natural geographical distribution of *Miscanthus* ranges from the tropics of the South Pacific and the subtropics of Southeast Asia to the cold temperate continental climate of northern China and eastern Russia (up to 50°N) (Clifton-Brown *et al.*, 2008; Sacks *et al.*, 2013), making it a useful model to study adaptation to chilling in C_4_ grasses. Relative to other C_4_ grasses, the chilling tolerance of some *Miscanthus* accessions is exceptional and well documented (Naidu *et al.*, 2003; Wang *et al.*, 2008; Friesen *et al.*, 2014; Głowacka *et al.*, 2014, 2015; Friesen, 2015; Pignon *et al.*, 2019). For instance, it was shown that leaves of *Miscanthus ×giganteus* grown at chilling (14 °C/11 °C day/night) or warm (25 °C/20 °C day/night) temperatures are able to maintain a very similar rate of CO_2_ assimilation across a range of temperatures between 5 °C and 38 °C; at the same time, chilling-grown maize loses ∼80% of CO_2_ assimilation in comparison with warm-grown plants (Naidu *et al.*, 2003). It was shown that *M.×giganteus*, after acclimatization to low temperatures, can achieve relatively high photosynthesis rates due to preservation of pyruvate orthophosphate dikinase (PPDK; Naidu *et al.*, 2003; Wang *et al.*, 2008) that plays a role in C_4_ photosynthesis. The ability of *M.×giganteus* to maintain PPDK protein content fairly unchanged under chilling treatment could thus contribute to maintaining relatively high CO_2_ assimilation abilities, reducing stress-related ROS formation and unregulated energy quenching. However, the mechanisms that protect photosynthesis during the short-term initial response to chilling are largely unknown, especially the role of NPQ. Importantly, in a temperate climate during leaf development of *Miscanthus*, at the end of April and the beginning of May, the typical temperature pattern consists of late chilling events interspersed by hot days, prohibiting the possibility of acclimatization to chilling (e.g. Supplementary Fig. S1). Therefore, the short-term initial response to chilling is more relevant to temperate climates. While *Miscanthus* is closely related to economically important C_4_ grasses, including maize, sorghum, and sugarcane, the genetic maps of *Miscanthus* and sorghum are highly linearly related and show high sequence conservation (Swaminathan *et al.*, 2012; Mitros *et al.*, 2020). Understanding the exceptional tolerance of *Miscanthus* is important for the development of this trait in non-chilling-tolerant relatives that play an important role in our economy.

Recently, and for the first time, we reported the dark operation of the xanthophyll cycle in a C_4_ plant during the initial short-term response to chilling. Specifically, the accessions from the northern distribution of the *Miscanthus* genus use the dark operation of the xanthophyll cycle to induce NPQ faster the following day (Haupt and Glowacka, 2024). Interestingly, the dark operation of the xanthophyll cycle was already seen at the end of a relatively short exposure to chilling during a single night. Here we hypothesize that dynamic adjustment of leaf pigment composition, including but not limited to xanthophylls, could contribute to mechanisms that protect photosynthesis during the initial short-term response to chilling. Considering ecological variation in the *Miscanthus* genus, we also further hypothesize that these changes in leaf composition are regulated by other stresses such as poor marginal soils that are present in many locations where *Miscanthus* naturally occurs. To test our hypotheses, we chose three *Miscanthus* accessions with contrasting chilling tolerance and overwintering abilities. We subjected these three *Miscanthus* accessions to chilling under dark and light conditions with or without nutrient limitations. Our results showed that the high-chilling-tolerant accession accumulates zeaxanthin and anthocyanins while reducing chlorophyll content during a night of chilling treatment when grown on the low-fertility soil in the growth chamber and field conditions. For the first time we showed that in *Miscanthus* the low-fertility soil was sufficient to induce an increase in dark accumulation of zeaxanthin in a high-chilling-tolerant accession; however, the addition of other stresses enhanced this phenomenon. The dynamic changes in pigments were positively linked to a gradient of stresses where changes in chlorophyl and zeaxanthin could be in part explained by up-regulation of the expression of enzymes involved in pigment anabolism and/or catabolism. Our work underlines that the co-existence of multiple stresses might have an ecological meaning to better prepare plants for chilling events.

## Materials and methods

### Plant material

Three accessions were chosen to represent a broad gradient of chilling tolerance. *Miscanthus sacchariflorus* ‘Robustus-Blumel’ (MsaRB; UI10-00009) shows exceptionally high chilling tolerance and originated from the north-eastern edge of the range of the species near the border of Russia and China (Głowacka *et al.*, 2015; Clark *et al.*, 2019). *Miscanthus ×giganteus* ‘Illinois’ (MxgI; UI10-00107), a nothospiecies derived from *M. sacchariflorus* and *M. sinensis* (Hodkinson and Renvoize, 2001; Hodkinson *et al.*, 2002; Friesen *et al.*, 2014; Głowacka *et al.*, 2014, 2015), collected in Yokohama (Japan), represents moderate chilling tolerance that is well documented (Naidu *et al.*, 2003; Wang *et al.*, 2008; Friesen, 2015; Pignon *et al.*, 2019). *Miscanthus sinensis* var. *condensatus* ‘Cosmo Revert’ (MsiCR; UI10-00014), which originated from Southern Japan, is characterized by low chilling tolerance and low overwintering abilities (Clark *et al.*, 2014; Głowacka *et al.*, 2014). All accessions were obtained as rhizomes from the *Miscanthus* Diversity Collection of the University of Illinois Urbana-Champaign. These rhizomes were multiplied vegetatively in the greenhouse using 6 liter pots (600c, Hummert International, Topeka, KS, USA), filled with growth medium (PROMIX BX Mycorrhizae, Premier Tech Home And Garden, PA, USA) with the addition of 40 g of granulated fertilizer (Osmocote 17-5-11, ICL Growing Solutions, Summerville, SC, USA). All physiological measurements and leaf samples were collected from the middle portion of the youngest fully expanded leaf (judged by the occurrence of ligule), avoiding the midrib.

### Growth chamber experiments

For growth chamber experiments, *Miscanthus* rhizomes were divided into 8–10 cm pieces and placed in trays containing holes (CN-FLXHD-X1, Greenhouse Megastore, Danville, IL, USA) filled with potting mix comprising 40% Canadian peat, 40% coarse vermiculite, 15% masonry sand, and 5% screened topsoil. After 3 weeks in the greenhouse (19/25 °C night/day), rhizomes started to sprout and were transferred to tree pots (MT49, Steue and Sons Inc., Tangent, OR, USA). For the experiment on fertile soil, tree pots were filled with a growth medium (PROMIX BX Mycorrhizae). The low-fertility soil treatment was induced by mixing growth medium (PRO-MIX BX Mycorrhizae) with sand in a ratio of 40:60 for the response of hyperspectral indexes to the combination of light and temperature, and in a ratio of 50:50 for the response of the Photochemical Reflectance Index (PRI) to the duration of growth. Analyses of soil chemical and physical properties (Ward Laboratories Inc., Kearney, NE, USA) for the fertile soil (100% PRO-MIX BX Mycorrhizae) and the low-fertility soil (50% PRO-MIX BX Mycorrhizae mixed with 50% of sand) are given in Supplementary Table S1. Pots were placed into a growth chamber (PGC20, Conviron, Manitoba, Canada) for 3 weeks with environmental conditions set at 20/25 °C night/day, 70% humidity, with a photoperiod of 12 h light maintained at 900 µmol m^−2^ s^−1^. Plants were watered twice a week and pots were randomly rotated in the growth chamber.

**Fig. 1.**
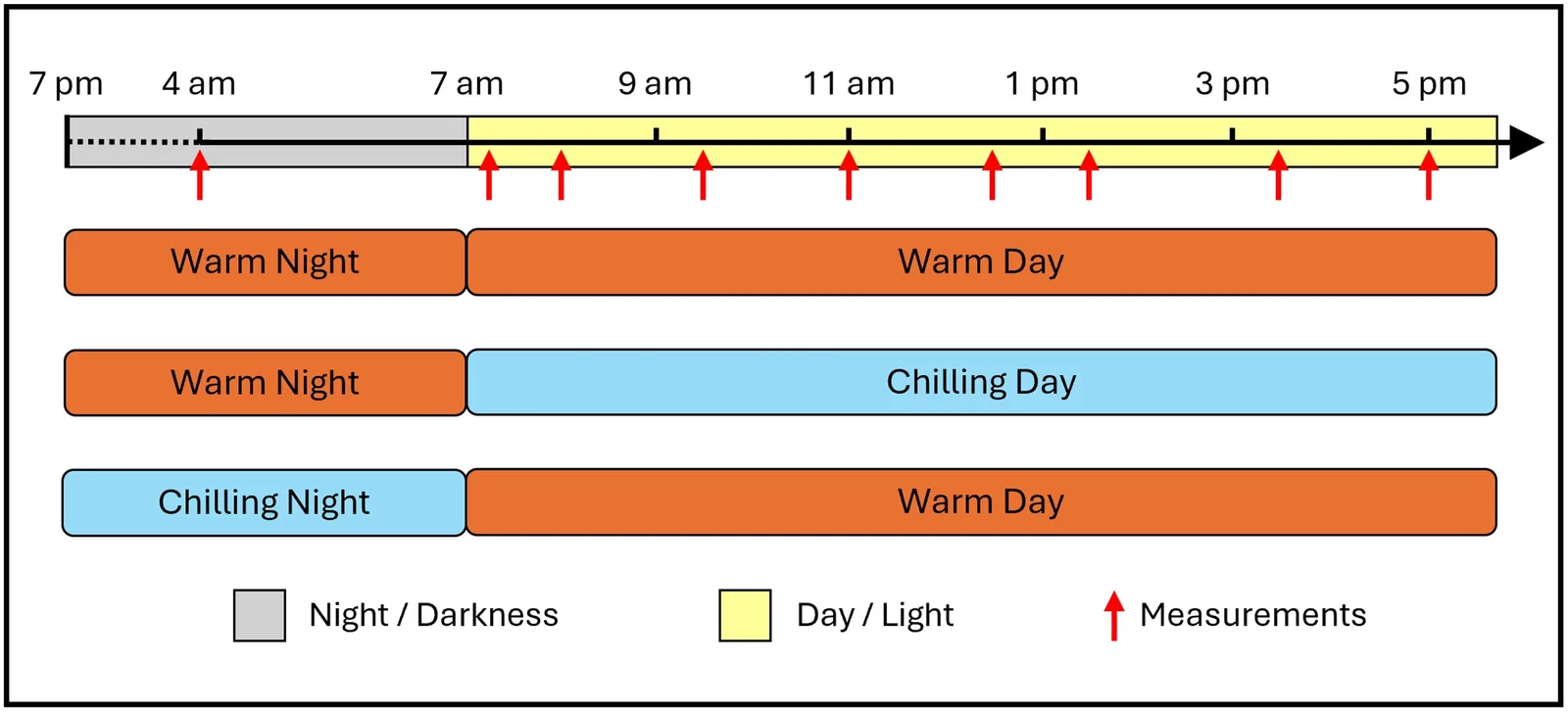
Schematic overview of the *Miscanthus* growth chamber experiments. Plants were grown in growth chambers under control conditions (20 °C/25 °C night/day) in fertile or low-fertility soil for 3 weeks and then subjected to three different temperature and light combinations: control (20 °C/25 °C night/day), warm night with chilling day (20 °C/10 °C night/day), or chilling night with warm day (5 °C/25 °C night/day). Photoperiod is indicated by the gray (night) and yellow (day) boxes. Temperature is indicated by orange (warm, 20 °C/25 °C night/day) and blue (chilling, 5 °C/10 °C night/day) boxes. Arrows indicate measurement points.

For experiments both in the fertile and low-fertility soil, after 3 weeks of growth, plants were subjected to the following temperature treatments (night/day): control (20/25 °C), chilling day preceded by warm night (20/10 °C), and chilling night followed by warm day (5/25 °C). All treatments started at the beginning of the night. Measurements were taken at the end of the first night (3 h before the start of the photoperiod; 04.00 h) and at the beginning of the first day (15 min of light; 07.15 h; Fig. 1). Diurnal measurements were done on the second and seventh day of treatment at seven time points: 1, 2.5, 4, 5.5, 6.5, 8.5, and 10 h following the start of the photoperiod corresponding to 08.00, 09.30, 11.00, 12.30, 13.30, 15.30, and 17.00 h.

### Field experiments

The field experiment at the University of Nebraska-Lincoln East Campus (Lincoln, NE; 40.829°N, 96.657°W) was established on 7 June 2019 on silt clay loam soil (Supplementary Table S1). Four blocks, with a completely randomized design were planted, each containing four single-plant plots of each of the three accessions, MsaRB, MxgI, and MsiCR. The field was organized in a 6×8 grid of plots with 1.5 m distance between plots. A single row of MxgI was planted around the fields as a border. Weeds were controlled mechanically and/or chemically as needed. The field was rain-fed (Supplementary Fig. S1) and not fertilized. Weather data were recorded at stations near the field location (Supplementary Fig. S1). Hyperspectral index measurements, samples for pigment and gene expression quantifications, and NPQ kinetics were collected on 13 September 2022.

### Hyperspectral indexes

For both growth chamber and field experiments, a leaf spectrophotometer (CI-710S SpectraVue; CID BiosScience Inc., Camas, WA, USA) was utilized to record leaf reflectance from a wavelength of 144 nm to 1334 nm. Measurements were taken using the manufacturer’s protocol. After instrument calibration, reflectance values were recorded within 5 s of clamping the leaf into the cuvette to minimize NPQ relaxation.

The PRI, negatively correlated with the accumulation of de-epoxidated xanthophyll pigments (Gamon *et al.*, 1997; Peñuelas *et al.*, 2011; Kováč *et al.*, 2018), the Anthocyanin Reflectance Index (ARI), positively correlated with anthocyanin content (Gitelson *et al.*, 2009), the Chlorophyll Content Index (CCI), positively correlated with total chlorophyll content (Gitelson and Merzlyak, 1997; Gitelson *et al.*, 2009), and the Carotenoid Content Index (CRI), positively correlated with the total carotenoid content (Gitelson *et al.*, 2009) were estimated from the measured leaf reflectance values using Equations (1–4), respectively:


(1 ) $$\mathrm{PRI}=\frac{R_{531}-\;R_{570}}{R_{531}\;+\;R_{570}}$$

(2 ) $$\mathrm{ARI}=\frac{1}{R_{550}}-\frac{1}{R_{700}}$$

(3 ) $$\mathrm{CCI}=\frac{R_{\mathrm{NIR}}}{R_{510}}$$

(4 ) $$\mathrm{CRI}=\frac{1}{R_{510}}-\frac{1}{R_{700}}$$

In all equations above, *R* stands for reflectance and the subscript indicates the wavelength for which the reflectance was measured. NIR stands for near infrared (from 760 nm to 800 nm).

### Xanthophyll cycle pigment, chlorophyll, and lutein quantification

HPLC analysis was carried to analyze the content of violaxanthin, antheraxanthin, zeaxanthin, lutein, Chl *a*, and Chl *b* in leaf samples. A weighted sample (20 mg) aliquot was extracted with acetone (100%; A9491; Fisher) in a 2 ml Eppendorf tube and homogenized using the Tissue Lyser II (Qiagen, Germantown, MD, USA) at 20 Hz for 10 min followed by centrifugation at 4 °C for 5 min at 16 000 *g* (centrifuge 5424; Eppendorf, Hamburg, Germany). The supernatant was collected and transferred to a HPLC vial. The pellets were re-extracted and supernatants were pooled together. The samples were dried down with a nitrogen stream (Microvap Microplate Evaporator 1181, Organomotion, Berlin, MA, USA). For LC separation, a Spherisorb S5 ODS1 column (4.6 mm × 250 mm, 5 µm; PSS830615, Waters, Milford, MA, USA) was used flowing at 1.2 ml min^–1^ at room temperature using a quaternary gradient on a UPLC Infinity II (Agilent, Santa Clara, CA, USA) equipped with a diode array detector (DAD). The gradient of the mobile phases A (0.1 M Tris base, pH 8.0; T1503, Sigma), B (100% acetonitrile; A955; Fisher), C (100% methanol; A456, Fisher), and D (100% ethyl acetate; E195; Fisher) was as follows: 14% A, 84% B, 2% C, 0% D to 0% A, 15% B, 60% C, 25% D over 15 min, then hold for 3 min before returning to initial conditions in 1 min. The detection of carotenoids was performed at 440 nm using the DAD. For quantification, an external standard curve of the carotenoids was used. Chl *a* and *b* were also measured and used for normalization of experimental variation.

### Anthocyanin quantification

Anthocyanin pigments were extracted following a protocol from Neff and Chory (1998). Four leaf discs were collected at 05.00 h using a hole punch (0.32 cm^2^) from a young fully expanded leaf in the field experiment. Samples were immediately snap-frozen in liquid nitrogen and stored at −80 °C until processing. Tissue was ground in liquid nitrogen and then mixed with 300 µl of methanol (34860; Sigma) with 1% HCl (320331; Sigma) to be stored overnight at 4 °C in the dark. Samples were diluted with 200 µl of Milli-Q H_2_O (Millipore Sigma, Burlington, MA, USA) and 500 µl of chloroform (25666; Sigma) to allow for phase separation of the water-insoluble chlorophylls and the water-soluble anthocyanins. Extracts were centrifuged at room temperature for 6 min at 21 000 *g*. The supernatant was collected and 320 µl were transferred to new tubes and diluted by adding 320 µl of a mixture of 60% methanol, 1% HCl, and 40% Milli-Q H_2_O. Diluted samples were transferred to a 96-well plate (167008; Thermo Fisher Scientific, Waltham, MA, USA) and absorbances were read at 530 nm and 657 nm using a microplate reader (BioTek Instruments, Inc., Winooski, VT, USA). Total anthocyanin content (ml g^–1^) was calculated using the following formula (Taghavi *et al.*, 2022):


(5 ) $$\mathrm{TA}=\;\frac{A_{530}-0.3\;\times A_{657}\times \;V}{m}$$

where TA is total anthocyanin content, *V* is the volume of extract in the well plate (ml), and *m* is the mass of the leaf sample in g; *A*_530_ and *A*_657_ stand for absorbance at 530 nm and 657 nm, respectively.

### Non-photochemical quenching kinetics

NPQ kinetics were investigated on leaf discs as previously described in Sahay *et al.* (2023). Leaf discs were collected using a hole punch (0.32 cm^2^), placed in a 96-well plate (781611; BrandTech Scientific, Essex, CT, USA), and covered with moist sponges. Plates were placed in a stay foam box with an ice pack, to keep leaf discs at chilling temperature, while transferring them from the collection point to the laboratory. Within 1 h after sample collection, plates were imaged using a modulated chlorophyll fluorescence imager (FluorCam FC 800-C, Photon Systems Instruments, Drasov, Czech Republic). First, the minimum (*F*_o_) and maximal (*F*_m_) fluorescence in the dark were imaged. Subsequently, the plates were subjected to 10 min of 2000 µmol m^−2^ s^−1^ followed by 10 min of darkness. Saturating flashes of 3200 µmol m^−2^ s^−1^ (provided by a cool white 6500 K light) were used in the light and dark periods to capture changes in steady-state (*F*_s_) and maximum (*F*_m_') fluorescence over time. Saturating flashes were provided at the following intervals (in minutes after light exposure began): 0, 0.263, 0.413, 0.747, 1.08, 2.08, 3.08, 4.08, 5.08, 6.08, 7.08, 8.08, 9.08, 10.08, 10.263, 10.413, 10.913, 11.913, 15.08, and 20.08. NPQ was calculated using Equation (6), assuming the Stern–Volmer quenching model (Bilger and Bjorkman, 1994).


(6 ) $$NPQ=F_{m}/F_{m}^{\prime }-1$$

The NPQ curves were fitted to a hyperbolic equation (Sahay *et al.*, 2023) in MATLAB (Matlab R2019b; MathWorks, Natick, MA, USA) to parametrize the rate of kinetics in the light and dark. The residual NPQ in the dark was calculated from fitting an exponential equation to the NPQ curve.

### Quantification of gene expression

Leaf discs were sampled from a young fully expanded leaf at 05.00 h in the field (end of the night) and the middle of the night (after 6 h of darkness) from control-grown plants under the growth chamber conditions (warm night and day on nutrient-rich soil, i.e. 100% PRO-MIX BX Mycorrhizae). After collection, both sample types were immediately shock-frozen in liquid nitrogen. For sample processing, leaf tissue was homogenized in liquid nitrogen to obtain a fine powder before total RNA extraction using a NucleoSpin RNA/Protein kit (740933; MACHEREY-NAGEL GmbH & Co., Duren, Germany). RNA extracts were then treated with the TURBO DNA-free kit (AM1907; Thermo Fisher) to remove the remaining genomic DNA. The cDNA was synthesized using a SuperScript III First-Strand Synthesis kit (18080051; Thermo Fisher). Quantitative reverse transcription–PCR (RT–qPCR; 1855201; BioRad CFX Connect Real-Time PCR Detection System, BioRad, Hercules, CA, USA) was performed using primers (Supplementary Table S2) designed to amplify gene sequences conserved across C_4_ grasses including *Miscanthus sinensis* v7.1, *Setaria italica* v2.2, *Setaria viridis* v4.1, and *Panicum vigatum* v5.1 (Joint Genome Institute). *Ubiquitin* (*UB*) and *Elongation factor-1-α* (*EF*) genes were used for normalization in the ΔΔCt method. From three to six biological replicates were analyzed per accession×treatment combination. The results of the two technical replicates were averaged before performing the statistical analysis.

### Statistical analysis

Normality and homoscedasticity were verified with the Shapiro–Wilk and Brown–Forsythe tests, respectively. If needed, the dependent variables were transformed (square root transformation) to meet model assumptions. The effects of ‘genotype’, and, when applicable, ‘treatment’, ‘time point’, ‘soil’, ‘day’, and interactions were tested with ANOVA (α=0.05). Data were fitted to a linear mixed-effects model with ‘genotype’, and, when applicable, ‘treatment’, ‘time point’, ‘soil’, and ‘day’ as fixed factors, and biological replicates as random factors (with the pot for the growth chamber experiment, or the plant for the field experiment, as a statistical unit). Differences between MsaRB or MxgI and MsiCR were tested with Dunnett’s post-hoc test, with MsiCR serving as a negative control. All statistical analyses were performed using R software (ver 4.2.1, (R Development Core Team, 2017), with packages lme4 (Bates *et al.*, 2015) for linear mixed-effect models, stats (R Development Core Team, 2017) for Shapiro–Wilk test, vGWAS (Shen *et al.*, 2012) for homoscedasticity, and DescTools (Signorell, 2023) for Dunnett’s test.

## Results

### The high-chilling-tolerant accession adjusts the content of multiple pigments in responses to stress intensity

**Fig. 2.**
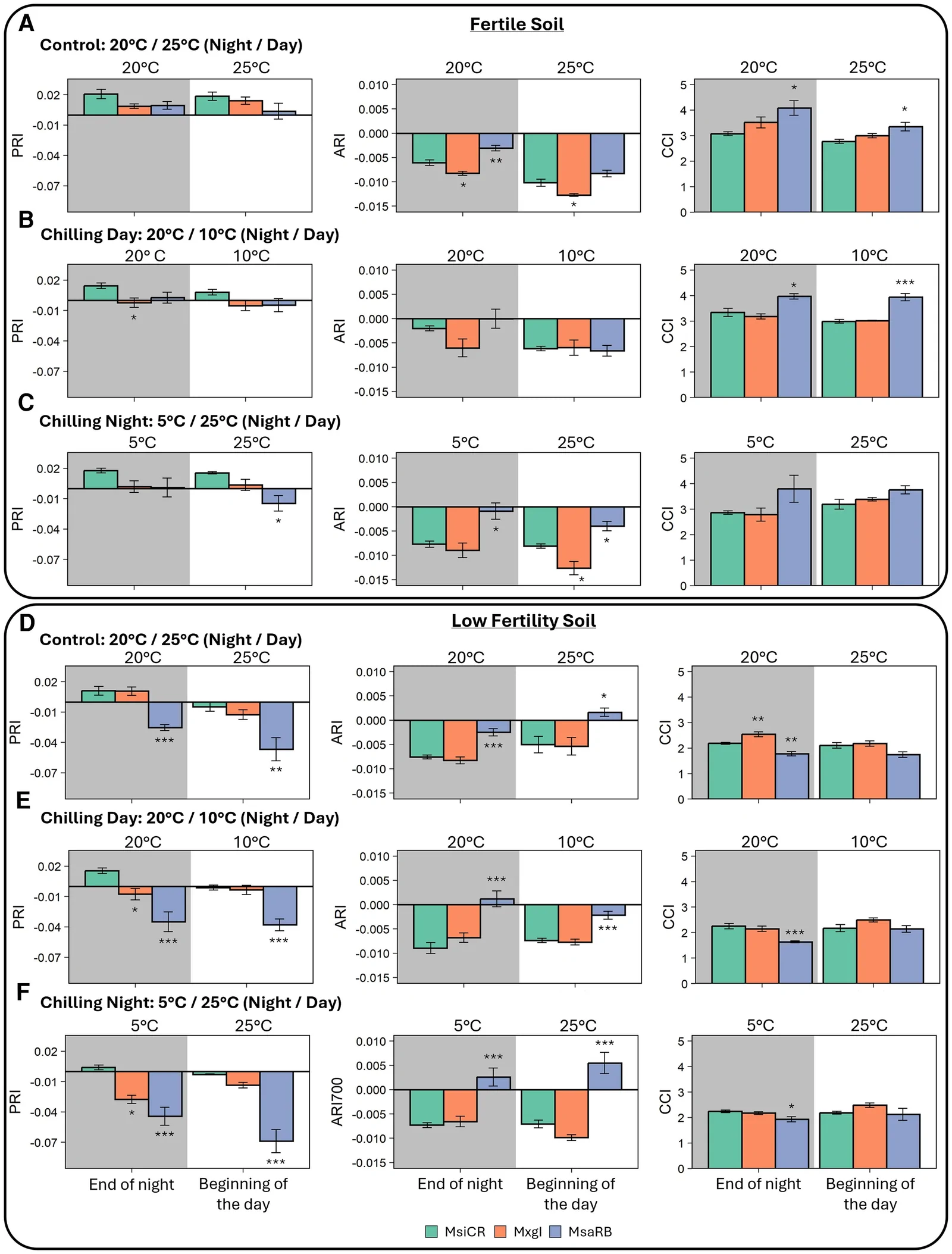
Response of hyperspectral indexes to soil fertility with a combination of light and temperature treatments in three *Miscanthus* accessions. Plants were grown in growth chambers under control conditions (20 °C/25 °C night/day) in (A–C) fertile or (D–F) low-fertility soil for 3 weeks and then subjected to three different temperature and light combinations: control (20 °C/25 °C night/day), warm night with chilling day (20 °C/10 °C night/day), or chilling night with warm day (5 °C/25 °C night/day). Hyperspectral indexes were measured on the youngest fully expanded leaf of *M. sinensis* (MsiCR; low chilling tolerance), *M.×giganteus* (MxgI; moderate chilling tolerance), and *M. sacchariflorus* (MsaRB; high chilling tolerance) at the end of the first night (9 h of night) and the beginning of the first day (15 min after the start of the photoperiod). Photochemical Reflectance Index (PRI; negatively correlated with de-epoxidation state of the xanthophylls), Anthocyanin Reflectance Index (ARI; positively correlated with anthocyanin content), and Chlorophyll Content Index (CCI; positively correlated with total chlorophyll content). All data are means (±SE) with *n*=4 biological replicates. Significant differences compared with MsiCR were determined using Dunnett’s test: *P≤0.05; **P≤0.01; ***P≤0.001.

To test the effect of a gradient of abiotic stresses, we investigated the pigment composition of the plant leaves grown under fertile and low-fertility soil with or without chilling. To separate the effect of chilling from the effect of high light, we investigated accessions at the end of the night or beginning of the day, where night and/or day could be chilled. To focus only on the early stage of plant response to chilling, the end of the first night (9 h of night) and the beginning of the following day (the first 15 min of light) were investigated. The effect of low-fertility soil was allowed to develop for a period of 3 weeks following rhizome sprouting (Fig. 1). In total, six combinations of temperature and light were tested. The leaf pigment composition was assessed via hyperspectral indexes to allow for repeated and high-throughput measurements (Fig. 2).

ANOVA showed that the accession had a highly significant effect on all measured hyperspectral indices (*P*≤0.0013; Supplementary Table S3). While the soil fertility had a highly significant effect on three out of four analyzed indexes (*P*<0.0001; Supplementary Table S3), the interaction between accession and soil fertility was highly significant for all indexes (*P*<0.0001; Supplementary Table S3). Interaction between soil fertility and temperature (*P*<0.0001; Supplementary Table S3) and between light and temperature (*P*≤0.05; Supplementary Table S3) had a significant effect on two indexes. Overall, the results show that the differences in indexes between the accessions varied significantly with changes in temperature, light, and soil fertility. The PRI decreased with increasing stress intensity and responded to temperature, light, and soil fertility, showing the most pronounced differences in plants grown on low-fertility soil under chilling night combined with warm day treatment. The ARI increased with increasing stress and responded primarily to soil fertility and then temperature, with the highest levels observed on low-fertility soil and then low temperature. The CCI decreased with lower soil fertility and was primarily influenced by soil conditions rather than temperature or light. The CRI was dependent on the species and showed no significant impact from any of the stress factors.

### Growth on fertile soil

On the fertile soil, PRI was generally the lowest in accessions with the highest or moderate chilling tolerance—MsaRB and MxgI—across all measured time points. However, the differences between accessions representing both extremes of chilling tolerance, MsaRB and MsiCR, became more prominent and significant with the increase in abiotic stress intensity (Fig. 2A versus C). After a warm night, no difference in PRI was observed between MsaRB and MsiCR under either warm or chilling day conditions (Fig. 2A, B). On the contrary, chilling night treatment combined with a warm day significantly decreased PRI by 2-fold in MsaRB compared with MsiCR (*P*<0.05; Dunnett’s two-way) (Fig. 2C). When grown on the fertile soil, MsaRB showed the highest anthocyanin content at most of the measured time points (Fig. 2A–C). Chilling night treatment with a combination of warm day increased ARI significantly by 9-fold (night) and 0.5-fold (day) in MsaRB compared with MsiCR (*P*<0.05; Dunnett’s two-way). Similarly, the differences in ARI between MsaRB and MsiCR became more prominent and significant with the increase of abiotic stress intensity (Fig. 2C). CCI mostly responded to genotype (*P*<0.0015) rather than chilling in combination with light or dark (Fig. 2A–C). CCI was generally the highest in the accession with the highest chilling tolerance, MsaRB, across all measured time points. MsaRB had significantly higher CCI than MsiCR (by 27% on average; *P*<0.05; Fig. 2A, B) during warm night, warm day preceded by warm night, and chilling day preceded by warm night. However, the differences between accessions representing the extremes of chilling tolerance became less pronounced and not significant with the increase of abiotic stress intensity due to a greater variation between biological replicates and stronger reduction of CCI in MsaRB than in MsiCR (Fig. 2C). Under the fertile soil conditions, CRI was significantly higher in MsaRB (98% on average; *P*<0.01) than in MsiCR in all tested combinations of temperature and light (Supplementary Fig. S2A–C). The accession with moderate chilling tolerance had an intermediate CRI value compared with the two other accessions.

### Growth on low-fertility soil

When grown on the low-fertility soil, the PRI was significantly the lowest in the accession with the highest chilling tolerance, MsaRB, at all measured time points (*P*<0.01; Fig. 2D–F). Interestingly, low soil fertility alone was able to induce significant differences in zeaxanthin formation between accessions at night. The magnitude of difference in PRI between the two accessions with the most contrasting chilling tolerance increased with stress intensification. PRI of MsaRB relative to MsiCR was 3.2-fold (warm night) to ≥24-fold lower for warm day preceded by chilling night and chilling night alone (Fig. 2D–F). On the low-fertility soil, MsaRB showed significantly the highest ARI, in all tested combinations of temperature and light (*P*<0.05; Fig. 2D–F); however, the effect of intensified abiotic stress in addition to low-fertility soil was less pronounced on ARI than on PRI. The changes between MsaRB and MsiCR ranged from 75% (control night) to 177% (warm day preceded by chilling night). In contrast to fertile soil experiments, under low-fertility soil MsaRB had the lowest CCI among all accessions without an obvious impact of temperature. While all accessions showed a decrease in chlorophyll content due to low soil fertility, the reduction in MsaRB was more severe than in the other two accessions. The significant differences observed between MsaRB and MsiCR were due to the soil fertility rather than the combination of temperature and light treatments. CRI was significantly higher in MsaRB than in MsiCR in most of the tested combinations of temperature and light under the low-fertility soil (Supplementary Fig. S2D–F). In the low-fertility soil in comparison with the fertile soil experiment, MsaRB showed an ∼30% reduction of CRI at night, both warm and chilling (Supplementary Fig. S2), while MsiCR had a fairly stable CRI on both fertile and low-fertility soil (0.025±0.01).

**Fig. 3.**
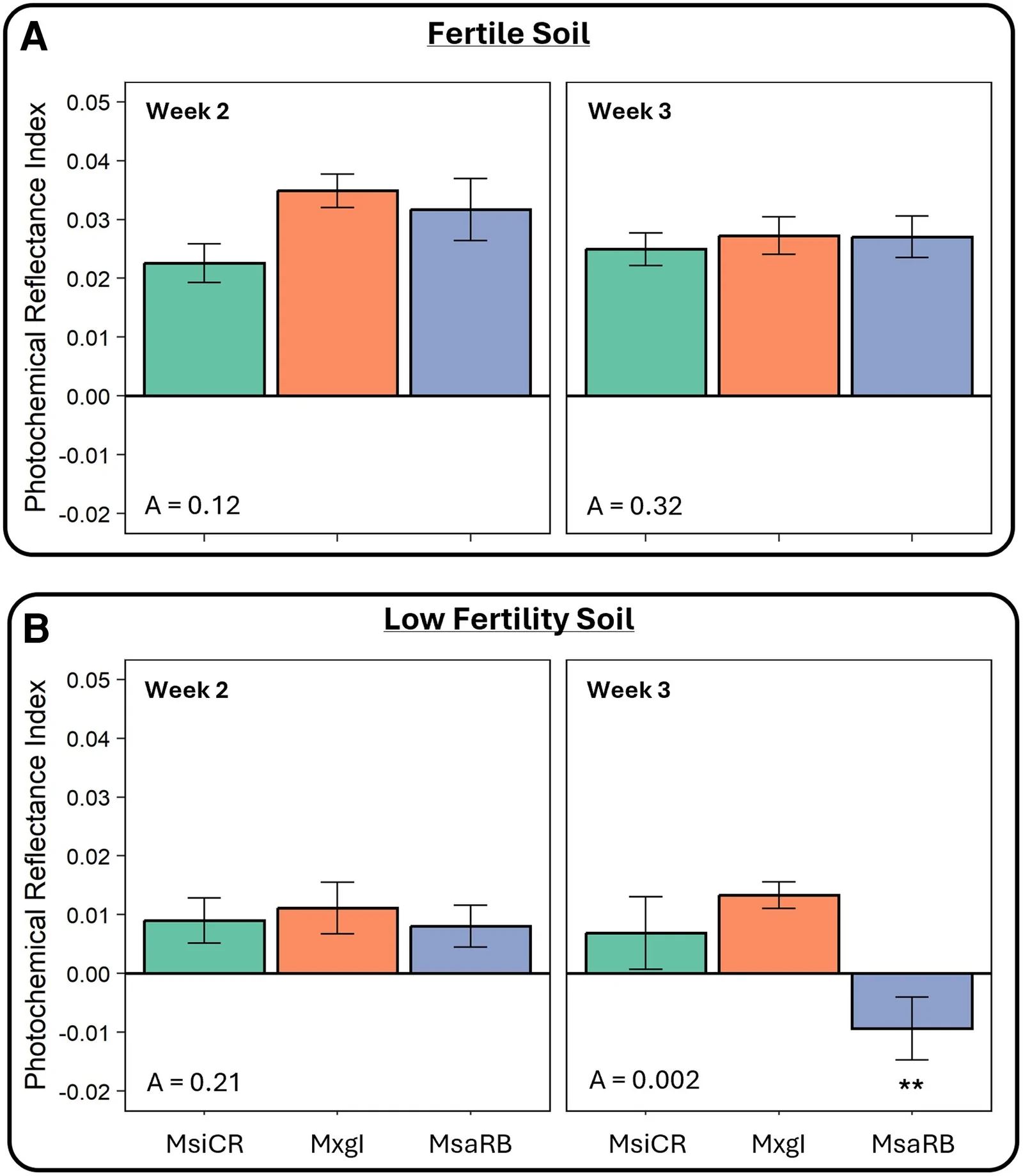
Response of the Photochemical Reflectance Index (PRI; negatively correlated with de-epoxidation state of the xanthophylls) to the duration of growth on two contrasting soil fertilities in three *Miscanthus* accessions. Plants were grown in growth chambers under control conditions (20 °C/25 °C night/day) in (A) fertile or (B) low-fertility soil for 3 weeks. Hyperspectral indexes were measured on the youngest fully expanded leaf of *M. sinensis* (MsiCR; low chilling tolerance), *M.×giganteus* (MxgI; moderate chilling tolerance), and *M. sacchariflorus* (MsaRB; high chilling tolerance) in the second and third week of growth at the end of the night (9 h of night). All data are means (±SE) with *n*=8 and *n*=7 biological replicates for (A) and (B), respectively. The *P*-values indicate the effect of accession (A) in the ANOVA. Significant differences compared with MsiCR were determined using Dunnett’s test: **P≤0.01.

Since soil fertility had a highly significant effect on the indexes investigated (*P*<0.001; ANOVA; Supplementary Table S3), we asked the question of how long it takes for these differences to develop. To answer this question, plants were grown on fertile and low-fertility soil (Supplementary Table S1) under warm night and warm day conditions for 3 weeks and the PRI, one of the indices most responsive to stress, was measured every other day. After 2 weeks of growth, there was no significant effect of accession on PRI in either of the soils (*P*≥0.12; Fig. 3). However, at the end of the third week, there was a significant effect of accession when grown on a low-fertility soil (*P*=0.002), with MsaRB showing significantly lower PRI than MsiCR (2.3-fold; *P*=0.0017). In contrast, on the fertile soil, accession continued to have no effect on PRI (*P*=0.3).

### Pigment composition shows varied levels of stability through the first 7 d of chilling

To assess diurnal variation in pigment composition in response to stress, hyperspectral indexes were measured between 08.00 h and 17.00 h on the seventh (Supplementary Figs S3, S5) and second (Supplementary Figs S4, S5) day of three treatments (warm day and night; warm day chilling night; and chilling day warm night) using the same plants as described above.

The accession, soil fertility, and treatment continued to have a strong significant effect (*P*<0.0001), while the time of day did not have a significant effect (*P*≥0.06) on measured indexes (ANOVA, Supplementary Table S4). Although the day of the experiment significantly affected the investigated indexes (*P*<0.0001), there was no significant interaction between accession and the day of the measurement except for PRI (*P*=0.03). When compared with the first day of treatment in light (Fig. 2), the accession differences in the pigment composition were mostly maintained when the treatment continued for 2 d and 7 d. For instance, PRI under fertile soil in chilling night/warm day treatment was significantly lower in MsaRB than in MsiCR throughout the seventh but not the second day of treatment (Supplementary Figs S3A–C, S4A–C). However, under low-fertility soil, strong significant differences between MsaRB and MsiCR were observed during the first day of treatments with chilling and maintained throughout days 2 and 7 (Supplementary Figs S3E–F, S4B–C). Under low-fertility soil conditions, the increase in ARI initially observed in MsaRB relative to MsiCR during a chilling day preceded by a warm night was not maintained at day 2 or day 7 (Supplementary Figs S3E, S4E). On the contrary, when MsaRB was grown in low-fertility soil and exposed to a warm day preceded by either a chilling night or a warm night, the increased ARI levels were maintained on the second and seventh day (Supplementary Figs S3F, S4F). The significantly higher chlorophyll content observed in MsaRB relative to MsiCR on the first day of two treatments under fertile soil conditions was maintained on the second and seventh day (Supplementary Figs S3A–B, S4A–B). Interestingly, none of the significant differences detected between MxgI and MsiCR on the first day of treatments was maintained on the next 6 d.

### Naturally occurring combination of stresses in the field intensifies pigment differences between accessions with contrasting chilling tolerance

**Fig. 4.**
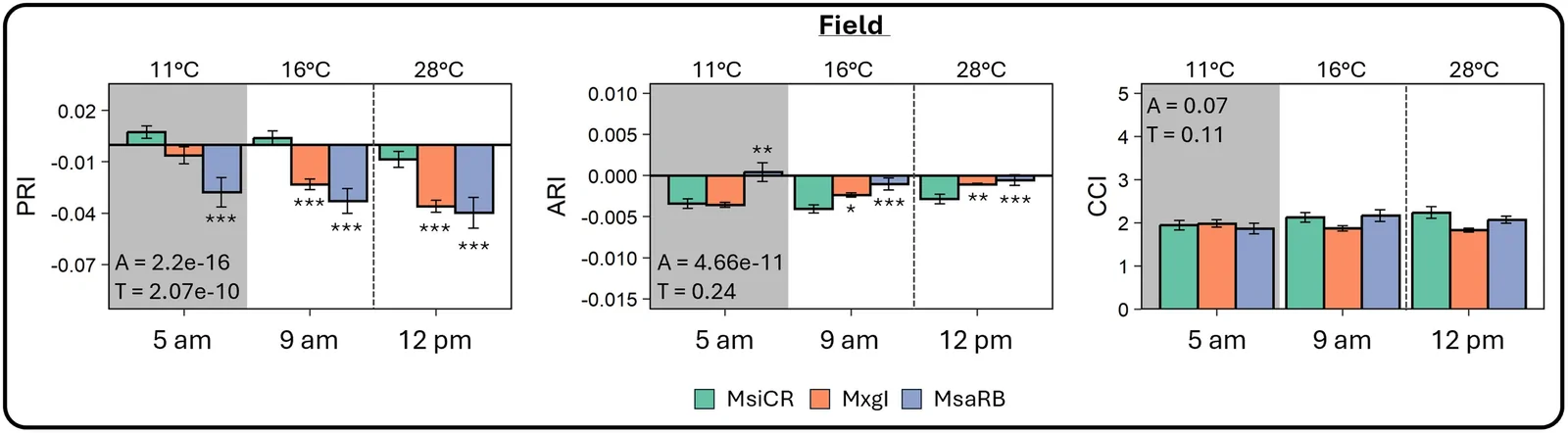
Response of hyperspectral indexes in three *Miscanthus* accessions grown in the field trial. Leaf reflectance was measured pre-dawn (05.00 h), mid-morning (09.00 h), and at noon (12.00 h) on the youngest fully expanded leaf of *M. sinensis* (MsiCR; low chilling tolerance), *M.×giganteus* (MxgI; moderate chilling tolerance), and *M. sacchariflorus* (MsaRB; high chilling tolerance) following the naturally occurring chilling event. The field was located at the University of Nebraska-Lincoln East Campus, Lincoln, NE (40.829 N, 96.657 W). The field was rain-fed, not fertilized (Supplementary Table S1), and temperatures overnight low and day high were 10 °C and 35 °C (for full weather data, see Supplementary Fig. S1). The Photochemical Reflectance Index (PRI; negatively correlated with the de-epoxidation state of the xanthophylls), Anthocyanin Reflectance Index (ARI; positively correlated with anthocyanin content), and Chlorophyll Content Index (CCI; positively correlated with total chlorophyll content). All data are means (±SE) with *n*=6 biological replicates. The *P*-values indicate the effect of accession (A) and time (T) in the ANOVA. Significant differences compared with MsiCR were determined using Dunnett’s test: *P≤0.05; **P≤0.01; ***P≤0.001

To investigate the response of pigment composition to a natural gradient of abiotic stresses, *Miscanthus* accessions with contrasting chilling tolerance were grown in a field trial without fertilization and under rain-fed conditions (Supplementary Fig. S1; Supplementary Table S1). Before measurements of hyperspectral indices, the trial experienced the first late summer chilling event, with the air temperature dropping to <10 °C (Supplementary Fig. S1). MsaRB had a PRI from 4.5- to 12-fold lower than MsiCR (*P*<0.001). Also, MxgI had a significantly lower PRI than MsiCR (*P*<0.001; Fig. 4). In comparison with MsiCR, MsaRB showed an increase in ARI by 112% at pre-dawn (*P*<0.01), by 75% at mid-morning (*P*<0.001), and by 82% at noon (*P*<0.001). MxgI had a significantly higher ARI than MsiCR (*P*<0.05; Fig. 4). The CCI of MsaRB was not significantly different from that of MsiCR (Fig. 4), while CRI was higher in MsaRB than in MsiCR by 46% (*P*<0.001) at mid-morning and noon (Supplementary Fig. S6).

**Fig. 5.**
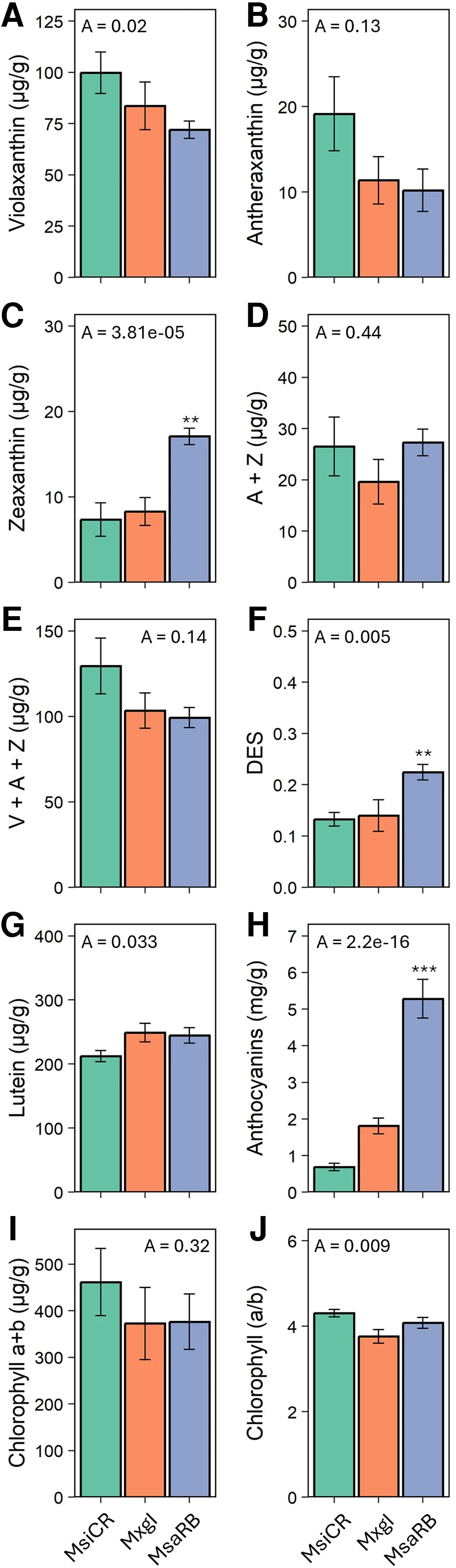
Pigment abundance in leaves of three *Miscanthus* accessions grown in the field trial after naturally occurring chilling. (A) Violaxanthin (V); (B) antheraxanthin (A); (C) zeaxanthin (Z); (D) sum of de-epoxidized xanthophyll cycle pigments (A+Z); (E) sum of xanthophyll cycle pigments (V+A+Z); (F) de-epoxidation state of the xanthophyll cycle {DES=[(0.5×A)+Z]/(V+A+Z)]; (G) lutein; (H) anthocyanins; (I) sum of Chl *a* and *b*; (J) ratio of Chl *a* and to Chl *b*. Pigments were quantified from tissue collected from the youngest expanded leaves of *M. sinensis* (MsiCR; low chilling tolerance), *M.×giganteus* (MxgI; moderate chilling tolerance), and *M. sacchariflorus* (MsaRB; high chilling tolerance) grown in the field (rain-fed and not fertilized) at the University of Nebraska-Lincoln East Campus (location: 40.829 N, 96.657 W). For the night of sampling, the overnight low temperature was 10 °C (for full weather data, see Supplementary Fig. S1). The *P*-values indicate the effect of accession (A) in the ANOVA. All data are means (±SE) with *n*=6 biological replicates. Significant differences compared with MsiCR were determined using Dunnett’s test: **P≤0.01; ***P≤0.001.

Since hyperspectral indexes offer only an approximation of pigment quantification, we molecularly quantified the pigment composition in the leaf samples from the field trial that were collected at pre-dawn in close time proximity to the collection of spectral measurements. To investigate further the conversion of xanthophyll cycle pigments, both epoxidated and de-epoxidated components of the cycle were analyzed. Lutein quantification was also performed since lutein was speculated to be the site of quenching in NPQ (H. Li *et al.*, 2020). The relative differences between accessions in leaf pigment composition assessed hyperspectrally (Fig. 4) agreed with the molecular quantification of pigments (Fig. 5). Higher PRI in MsaRB relative to MsiCR in pre-dawn samples was related to a 131% increase in zeaxanthin content (Fig. 5E, P<0.01). The violaxanthin, antheraxanthin, and xanthophyll cycle pigment pool was not significantly different between MsaRB and MsiCR (Fig. 5A, C, D). As a consequence of higher zeaxanthin levels in combination with a similar xanthophyll cycle pigment pool, a higher de-epoxidation state (DES) was observed in MsaRB in comparison with MsiCR (+69%, *P*<0.01; Fig. 5F). Quantification of lutein showed no significant increase in MsaRB relative to MsiCR, which agreed with the CRI (Fig. 5G; Supplementary Fig. S6). The anthocyanin content was 8-fold higher in MsaRB than in MsiCR (*P*<0.001), which closely agreed with the estimation via the ARI (Figs 2, 5H). In agreement with the CCI (Fig. 4), the total chlorophyll content and Chl *a*/*b* ratio remained the same among accessions (Fig. 5I, J).

### Transcriptional regulation of enzymes associated with pigment anabolism and catabolism supports the dynamic adjustment in leaf pigment composition

To probe the mechanism that could allow for the dynamic changes observed in leaf pigment composition, we quantified the transcript abundance of enzymes involved in conversion of xanthophyl cycle pigments, violaxanthin de-epoxidase and (VDE) and zeaxanthin epoxidase (ZEP), together with PsbS, a crucial protein for NPQ that senses the pH changes on the lumen side of the thylakoid membrane. Transcript abundance was also quantified for genes encoding enzymes involved in the late step of anthocyanin synthesis, dihydroflavonol 4-reductase (DFR), and chlorophyll anabolism, glutamyl-tRNA reductase (GluTR), and catabolism, magnesium-dechelatase (NYE). To allow direct comparison, transcripts were quantified from the same leaves used for hyperspectral measurements and pigment composition assay. Since in the field it is difficult to obtain samples equivalent to control conditions, we compared field data with growth-chamber-grown plants under well-watered and nutrient-rich soil conditions that were sampled in the middle of a warm night.

**Fig. 6.**
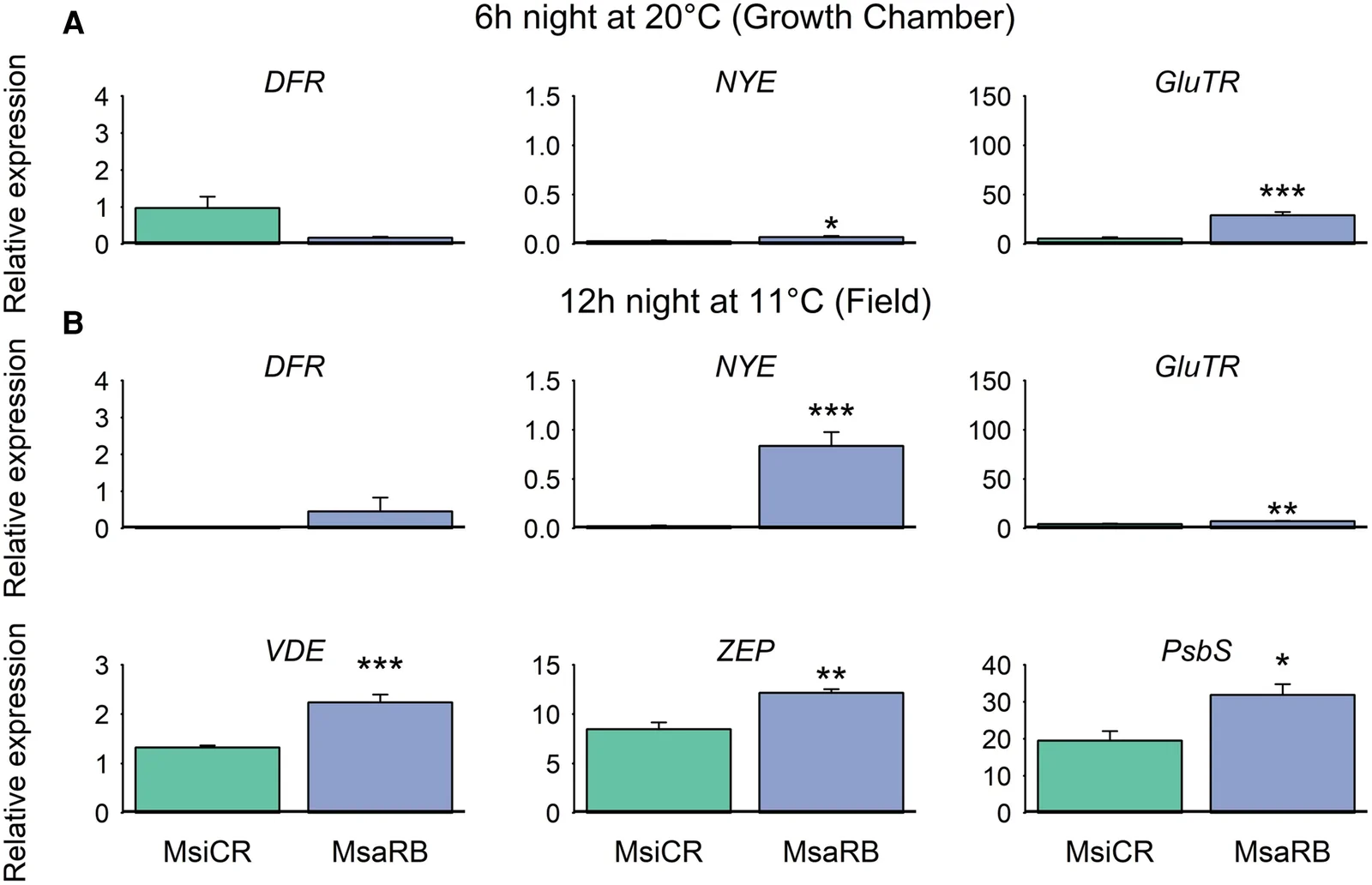
Transcript abundance of the anthocyanin biosynthesis (*DFR*), chlorophyll metabolism (*GluTR* and *NYE*), and three key NPQ (*VDE*, *ZEP*, and *PsbS*) genes in leaves of the two most contrasting *Miscanthus* accessions. Transcript abundances were quantified from tissue collected from the youngest expanded leaves of *M. sinensis* (MsiCR; low chilling tolerance) and *M. sacchariflorus* (MsaRB; high chilling tolerance) grown (A) for 6 weeks in growth chambers under control conditions (20 °C/25 °C night/day) in fertile soil; (B) in the field (rain-fed and not fertilized) at the University of Nebraska-Lincoln East Campus (location: 40.829 N, 96.657 W). For the night of sampling in the field, the overnight low temperature was 10 °C (for full weather data, see Supplementary Fig. S1). All data are means (±SE) with *n*=3–6 biological replicates. Gene expression is normalized to the *Ubiquitin* and *Elongation factor-1-α* genes. Significant differences compared with MsiCR were determined using Dunnett’s test: *P≤0.05; **P≤0.01; ***P≤0.001.

In the field at the end of a chilling night, transcripts of both genes responsible for xanthophyll cycle operation, *VDE* and *ZEP*, were significantly higher in MsaRB relative to MsiCR by 69% (*P*=0.00015) and 43% (*P*=0.0037), respectively (Fig. 6). Transcript abundance of *PsbS* was also higher in MsaRB than in MsiCR (+63%, *P*=0.0157) as was that of *DFR* (16-fold), without significant differences between genotypes (Fig. 6). At the same time, transcripts levels of genes involved in chlorophyll metabolism, *GluTR* and *NYE*, were respectively 1.6- and 39-fold higher (*P*=0.0036 and *P*=0.00034, respectively) in MsaRB than in MsiCR. Furthermore, relative to control conditions, the *GluTR* transcript abundance was four times lower in MsaRB but remained stable in MsiCR (<9% difference; Fig. 6). The *NYE* transcript abundance was 12-fold higher in MsaRB but only 2-fold higher in MsiCR under control conditions.

### Stress-induced formation of zeaxanthin in the night leads to a higher NPQ induction rate on the following day

**Fig. 7.**
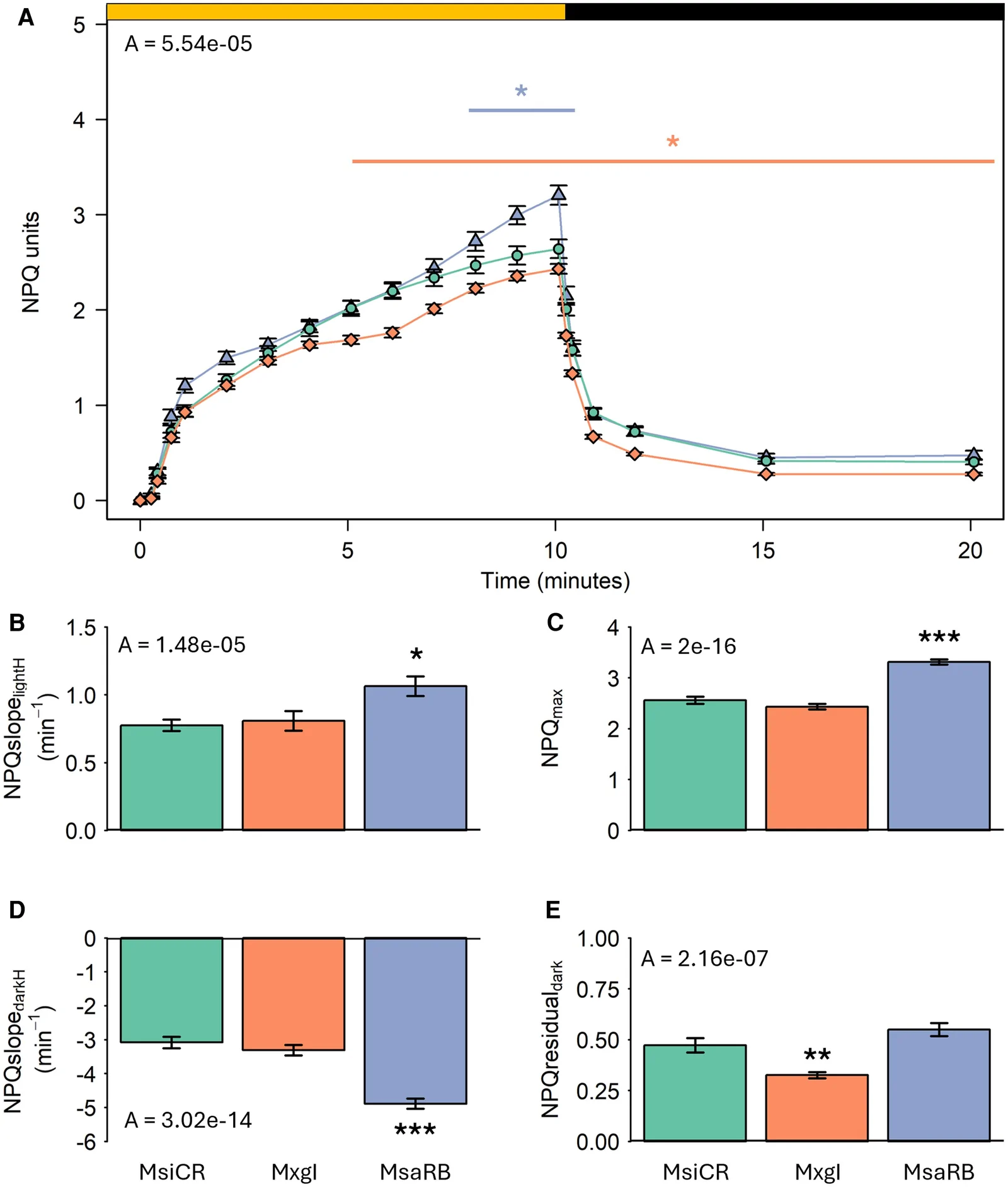
Response of non-photochemical quenching (NPQ) for naturally occurring chilling in three *Miscanthus* accessions grown in the field trial. Leaf samples for NPQ kinetics assay were collected pre-dawn (05.00 h) from the youngest fully expanded leaf of *M. sinensis* (MsiCR; low chilling tolerance), *M.×giganteus* (MxgI; moderate chilling tolerance), and *M. sacchariflorus* (MsaRB; high chilling tolerance) grown at the University Nebraska-Lincoln East Campus, Lincoln, NE (40.829N, 96.657W). The field was rain-fed, not fertilized (for soil analyses, see Supplementary Table S1) and overnight temperature was 10 °C (for full weather data, see Supplementary Fig. S1). (A) NPQ induction (10 min of light) and relaxation (10 min of dark) curves; (B) rate of NPQ induction in light (NPQslope_lightH_); (C) maximum NPQ in light (NPQ_max_); (D) rate of NPQ induction in the dark (NPQslope_darkH_); and (E) non-relaxed NPQ at the end of the dark period (NPQresidual_dark_). All data are means (±SE) with *n*=6 biological replicates. The *P*-values indicate the effect of accession (A) in the ANOVA. Significant differences compared with MsiCR were determined using Dunnett’s test: *P≤0.05; **P≤0.01; ***P≤0.001. In (A), the blue and orange lines above the curves represent significant difference between MsaRB (blue) or MxgI (orange) and MsiCR for multiple time points.

Since xanthophyll cycle pigments are closely involved in the NPQ mechanism and showed, in our study, one of the most prominent changes between investigated accessions, we measured the effect of these changes on the NPQ kinetics. To allow direct comparison, NPQ (Fig. 7) was investigated in leaf samples from the field-grown plants collected at pre-dawn on the same day as hyperspectral indexes (Fig. 3), samples for pigment quantification (Fig. 5), and gene expression (Fig. 6). The rate of NPQ induction in light calculated from the slope of the induction curve increased by 38% in MsaRB compared with MsiCR (Fig. 7B; *P*<0.05). The maximum level of NPQ achieved at the end of 10 min of light was also significantly higher (29%; *P*<0.001) in MsaRB relative to MsiCR (Fig. 7C).

## Discussion

Crops with improved chilling tolerance could allow early spring planting or regrowth to facilitate longer biomass accumulation, extend the light-harvesting period, and increase the use of early-season soil moisture. Here we showed that the *Miscanthus* accession from the north-eastern edge of this genus distribution previously documented as highly chilling tolerant (Sacks *et al.*, 2013; Głowacka *et al.*, 2014) accumulates zeaxanthin at the end of a chilling night in a stress-dependent fashion (Figs 2, 4). In contrast, the accession from warmer locations—such as Southern Japan—characterized by low chilling tolerance (MsiCR; Clark *et al.*, 2014; Głowacka *et al.*, 2014) shows very limited, if any, zeaxanthin accumulation in response to environmental conditions. The dark accumulation of zeaxanthin, a crucial pigment for NPQ, would increase the speed of photoprotection (Demmig-Adams, 1990) the following morning. Indeed, when we inspected the NPQ response to the light in plants grown in the field under a combination of naturally occurring stresses with addition of a chilling night, we observed a zeaxanthin content 131% higher in MsaRB relative to MsiCR at the end of the night, which translated into an NPQ induction 38% faster in light (Fig. 7). Zeaxanthin can allosterically help in PsbS interaction with minor antenna proteins that is needed for conformational changes to occur in the light-harvesting complexes to achieve full NPQ potential (Betterle *et al.*, 2009; Correa-Galvis *et al.*, 2016; Sacharz *et al.*, 2017). MsaRB, with a higher zeaxanthin concentration at the end of the chilling night, also showed a 29% higher NPQmax level compared with MsiCR. This NPQ phenotype probably contributes to adaptation to chilling and sunny mornings that follow chilling nights, where faster and more sustainable NPQ would protect from oxidative stress. Consistent with this postulation, faster photosynthesis recovery was reported for MsaRB in comparison with negative controls after the days of chilling treatment (Głowacka *et al.*, 2014). The conversion of violaxanthin to zeaxanthin or zeaxanthin retention in the absence of light has been previously shown in organisms that are adapted to extremely challenging environments such as evergreen trees during winter (Demmig-Adams, 1990; Brueggemann *et al.*, 2009; Fernandez-Marin *et al.*, 2018). In response to abiotic stresses MxgI, that is a gold standard for chilling tolerance in the *Miscanthus* genus (Hodkinson and Renvoize, 2001; Hodkinson *et al.*, 2002; Friesen *et al.*, 2014; Głowacka *et al.*, 2014, 2015) collected in Yokohama (Japan) and which represents moderate chilling tolerance that is well documented (Naidu *et al.*, 2003; Wang *et al.*, 2008; Friesen, 2015; Pignon *et al.*, 2019), showed a less consistent and lower extent of adjustment of pigment composition than MsaRB. For instance, in the growth chamber experiment, when grown on the low-fertility soil with a combination of chilling night, MxgI showed 8-fold higher zeaxanthin accumulation and lack of significant changes in anthocyanins and chlorophylls relative to MsiCR (Fig. 2F). In the same conditions, MsaRB relative to MsiCR showed 24 times higher zeaxanthin content in addition to a significant increase in anthocyanins and a significant decrease in chlorophylls. Somehow, the intermediate response of MxgI relative to both MsaRB and MsiCR is what we would expect from the interspecific origin of MxgI which is a hybrid between *M. sacchariflorus* and *M. sinensis.* While the exact parental accessions of MxgI are unknown, it is very unlikely that one of them is MsaRB due to their different geographical origins (Hodkinson and Renvoize, 2001; Hodkinson *et al.*, 2002; Sacks *et al.*, 2013), which further could explain why MxgI, even as a chilling-tolerant accession, does not adjust its pigment composition with a similar pattern to MsaRB.

Interestingly, in our study, low soil fertility alone increased zeaxanthin content by 3.2-fold at the end of the night in the high-chilling-tolerant accession (MsaRB) versus the low-chilling-tolerant accession (MsiCR) (Fig. 2D, E; Supplementary Fig. S2). Furthermore, when MsaRB plants were grown on low-fertile soil, they needed 3 weeks to show the increase in dark accumulation of zeaxanthin (Fig. 3B). While mixing fertile soil with sand in a 1:1 ratio indeed significantly reduced the content of important nutrients (nitrate, phosphorus, potassium, calcium, magnesium, sodium, and sulfate; Supplementary Table S1), it also significantly affected other characteristics of soil, including a significant reduction in organic matter and change in soil texture from sandy loam to sandy, what could further reduce nutrient availability by holding less water and salts. In agreement with our observations on the biophysical attributes of leaves, da Costa *et al.* (2019) detected noticeable morphological changes on low-fertility soil in *Miscanthus* in week four of treatment. While morphological changes were not recorded in the third week by da Costa *et al.* (2019), it is very likely that more subtle changes such as those detectable with hyperspectral images happen earlier. A single chilling night in combination with low soil fertility further increased the differences in zeaxanthin content by 12-fold in high- versus low-chilling tolerant accessions. These results underline that, in MsaRB, the dark induction of NPQ might be ubiquitous but works on different time scales depending on the abiotic stress, and that the co-occurrence of stresses further enhances this dark regulation. Importantly, chilling at the beginning of the day increased zeaxanthin formation to a lesser extent than chilling at night (Fig. 2A versus B, and E versus F). Dark accumulation of zeaxanthin can serve as an ecological adaptation to locations with poor soils experiencing multiple stresses, including chilling temperatures (Sacks *et al.*, 2013), where low temperature at night with low soil fertility will trigger anticipation and preparedness for swift photoprotection in the coming morning. In comparison with other C_4_ crops, some *Miscanthus* accessions can withstand chilling and drought well, while still producing high biomass yield on marginal land (e.g. Kalinina *et al.*, 2017).

The total pool of xanthophylls and antheraxanthin under field stress conditions were not significantly different among *Miscanthus* accessions, indicating that the difference in zeaxanthin level between MsaRB and MsiCR is mostly due to conversion of violaxanthin to zeaxanthin rather than *de novo* zeaxanthin synthesis (Fig. 5). Meta-analysis of leaf pigment composition in 272 datasets (species×treatment) showed that the total amount of xanthophyll cycle pigments (VAZ) is highly responsive to the environmental stresses, and that among eight of the analyzed stresses, only chilling, cold, and heat significantly increased VAZ (Esteban *et al.*, 2015). Indeed, *M.×giganteus*, when allowed to grow and develop the leaves at 10 °C, increased the VAZ content by 54% relative to plants grown at 25 °C (Farage *et al.*, 2006). However, we previously showed that a single chilling night did not significantly change the VAZ pool in any of the *Miscanthus* accessions ranging from high- to low-chilling tolerant, although zeaxanthin content significantly increased only in high-chilling-tolerant accessions, including MsaRB (Haupt and Glowacka, 2024). These differences suggest that MsaRB, as well as other accessions from the northern distribution of the *Miscanthus* genus, might develop unique mechanisms to regulate the xanthophyll pigment cycle during short chilling episodes compared with when they are allowed to adapt for longer. Previously, we did not observe significant differences between MsaRB, MxgI, and MsiCR in transcript abundance of *VDE*, *ZEP*, and *PsbS* genes at the end of a warm or a chilling night when grown on fertile soil in the growth chamber, and we suggested post-transcriptional up-regulation of *VDE*, allowing for zeaxanthin formation under a night of chilling treatment (Haupt and Glowacka, 2024). However, under multiple stresses, in a non-fertilized (Supplementary Table S1) rain-fed field (Supplementary Fig. S1) after one of the first chilling nights (Supplementary Fig. S1) of the late summer, a significant increase in *VDE* transcript abundance was detected in MsaRB relative to MsiCR (Fig. 6). While *Miscanthus* has relatively low nutrient requirements (Cadoux *et al.*, 2012), the yearly collection of biomass removes essential nutrients from the soil, particularly nitrogen, leading to the recommendation of *Miscanthus* field fertilization with 50–101 kg nitrogen ha^–1^ (Shield *et al.*, 2014). In our 3-year-old field trial, the soil nitrate content was 15 kg ha^−1^, which is below the recommended *Miscanthus* fertilization rate, suggesting that our plants experienced a limitation of this essential nutrient. In addition to *VDE* transcript change in the field-grown plants after a chilling night, *ZEP* transcript abundance also significantly increased, while *PsbS* followed the same trend (Fig. 6). A higher abundance of *VDE* and *ZEP* in MsaRB could lead to a faster NPQ induction and relaxation, while increased *PsbS* could compensate for the xanthophyll cycle acceleration (Kromdijk *et al.*, 2016).

In our study, the high-chilling-tolerant accession showed a stress-dependent increase in anthocyanin content, as observed for zeaxanthin, while it remained relatively stable in the low-chilling-tolerant accession (Figs 2, 4). Anthocyanins play a role in photoprotection through their antioxidative properties and by absorbing yellow, green, and blue components of visible light which reduces PSII photoinhibition. In contrast to NPQ, anthocyanin-mediated photoprotection occurs outside of the chloroplast and particularly in stress conditions, including nutrient deficiency (Lea *et al.*, 2007; Zhang *et al.*, 2017; Meng *et al.*, 2020), chilling (Carmona *et al*., 2017), and high irradiance combined with low temperature (Pietrini *et al.*, 2002; Zhang *et al.*, 2018). An increasing body of research supports the idea of anthocyanins mostly functioning in photoprotection as a light screen reducing the quantity and modifying the quality of light that reaches the chloroplasts (e.g. Zheng *et al.*, 2021). In some species under changing seasonal conditions, anthocyanins compensate for low NPQ (Zhu *et al.*, 2018) and their accumulation alone cannot meet the needs of young leaves for photoprotection (Yu *et al.*, 2023). In our study, both NPQ and anthocyanins rather work in tandem. The regulation of anthocyanin biosynthesis is complex and involves multiple levels of transcriptional and translational regulation, as well as post-translational modification (Shi *et al.*, 2023). While *DFR*, an anthocyanin biosynthesis gene, was shown to be transcriptionally up-regulated in response to abiotic stresses (Xu *et al.*, 2014), we found only a trend for the *DFR* transcript level to be 16-fold higher in MsaRB than in MsiCR (Li *et al.*, 2012; Jiang *et al.*, 2022). The detected mismatch in MsaRB between the highly significant increase in total anthocyanin content (Fig. 5) and the non-significant increase in *DFR* transcript levels (Fig. 6) could be explained by an expressional up-regulation of anthocyanin synthesis happening at the beginning of a chilling night rather than at the end. Indeed, it was shown in apple that anthocyanin transcriptional up-regulation is induced early during a chilling event, with a peak observed after 1 h which then gradually decreases over 12 h (Jiang *et al.*, 2022). Furthermore, anthocyanins are quite stable in the acid–base environment of plant vacuoles where they are stored (Wang *et al.*, 2024) and can therefore subsist for a much longer time.

In our study, the low soil fertility reduced the total chlorophyll content in all accessions (Fig. 2), which was previously documented in other species (Baker *et al.*, 2023; Sahay *et al.*, 2024a, b). On the other hand, for none of the accessions did the chilling night alone change the total chlorophyll content relative to control warm conditions, whether using fertile (Fig. 2A versus C) or low-fertility soil (Fig. 2D versus F). Esteban *et al.* (2015), by consolidating 155 datasets (species×treatment), showed that salt stress significantly increased total chlorophyll content, in contrast to drought, chilling, and ozone stresses which significantly reduced their content. Continued growth of MxgI under 14 °C or 10 °C significantly reduced total chlorophyll content by 36% and 55%, respectively, in comparison with control conditions (Farage *et al.*, 2006). It is interesting to note that in our study on fertile soil, the high-chilling-tolerant accession had the highest total chlorophyll content, while showing the lowest content on low-fertility soil. This observation suggests that under favorable conditions the high-chilling-tolerant accession might absorb more light than the low-chilling-tolerant accession. However, under stress conditions, it reduces its chlorophyll content more strongly, lowering light absorption that can further contribute to photoprotection. In agreement with this observation, under chilling rain-fed non-fertilized conditions in the field, MsaRB showed a significant increase in the transcript abundance of the chlorophyll catabolic enzyme NYE relative to control conditions (*P*=0.00021), while in MsiCR its abundance remained relatively unchanged. Furthermore, significant changes in the transcript abundance of the chlorophyll synthesis gene, *GluTR*, were not observed between control and field conditions in any of the analyzed accessions. The chlorophyll content depends on the relative rate of chlorophyll anabolism and catabolism, while plant fitness depends on the dynamic regulation of chlorophyll levels in response to environmental changes (Wang *et al.*, 2020). Interestingly, our previous study of natural variation of NPQ in sorghum under nitrogen stress identified *NYE* to be significantly associated with multiple NPQ kinetic traits (Sahay *et al.*, 2024b). This finding further underlines that the fine-tuning of multiple pigments adjusts photoreaction to a changing environment and/or in response to abiotic stresses.

## Conclusion

A high-chilling-tolerant accession of *Miscanthus* fine-tunes pigment content through diversified means to adjust and achieve the optimal level of photoprotection. The dark accumulation of zeaxanthin increased the induction speed and level of NPQ the following morning. The observed increase in anthocyanin content can contribute to modification of the quality and reduces the quantity of light that reaches the chloroplasts, while the reduction in chlorophyll can reduce light absorption via antenna complexes. The high-chilling-tolerant accession used all these tools to acclimate to changing seasonal conditions such as chilling and continuing stress such as low soil fertility. Co-occurrence of stresses in our study enhances the magnitude of pigment changes. In many regions in the northern hemisphere, the future climate is expected to have warmer springs with more frequent and severe chilling events together with dryer summers (Cohen *et al.*, 2018; Soureshjani *et al.*, 2019; Chamberlain *et al.*, 2021; IPCC, 2021; Hu *et al.*, 2022). At the same time, the global population is expected to rise, leading to an increase in demand for agricultural products. Investigation of the dynamic changes in pigment composition can generate new approaches to engineering crops with higher resilience to extreme weather events.

## Supplementary Material

erag236_Supplementary_Data

## Data Availability

All data supporting the findings of this study are available within the paper and within its supplementary data published online.

## Supplementary data

The following supplementary data are available at *JXB* online

Fig. S1. Daytime high and overnight low temperatures (°C), along with daily precipitations (cm) collected for the field trial.

Fig. S2. Response of the Carotenoid Reflectance Index (CRI; indicative of carotenoid content) to soil fertility with the combination of light and temperature treatments in three *Miscanthus* accessions.

Fig. S3. Diurnal response of hyperspectral indexes on day 2 of three light and temperature treatments for contrasting soil fertilities in three *Miscanthus* accessions.

Fig. S4. Diurnal response of hyperspectral indexes on day 7 of three light and temperature treatments for contrasting soil fertilities in three *Miscanthus* accessions.

Fig. S5. Diurnal response of the Carotenoid Reflectance Index (CRI; indicative of carotenoid content) on days 2 and 7 of three light and temperature treatments for contrasting soil fertilities in three *Miscanthus* accessions.

Fig. S6. Response of CRI in three *Miscanthus* genotypes grown in the field trial.

Table S1. Composition analysis of soil samples.

Table S2. Primer sequences used in RT–qPCR.

Table S3. ANOVA for the first day of treatments.

Table S4. ANOVA for two diurnals on the second and seventh day of treatments.

Table S5. RT–qPCR cycle numbers.
