# Supplementary material for: Combination of abiotic stresses accelerates photoprotection in exceptionally chilling-tolerant C4 grass by fine-tuning the content of multiple pigments
Source: J Exp Bot. 2026 May 21;77(17):5754–70. doi: 10.1093/jxb/erag236 (PMC13577770; doi:10.1093/jxb/erag236)
Supplement: Supplementary file 1 [file erag236_supplementary_data.pdf]

Combination of abiotic stresses accelerates photoprotection in exceptionally chilling tolerant C4 grass by fine tuning content of multiple pigments

Benjamin Turc, Asha Kumari, Maria Carter, Katarzyna Glowacka

Fig. S1. Daytime high and overnight low temperatures (°C), along with daily precipitations (cm) collected for the field trial.

Fig. S2. Response of the Carotenoid Reflectance Index (CRI; indicative of carotenoid content) to soil fertility with the combination of light and temperature treatments in three Miscanthus accessions.

Fig. S3. Diurnal response of hyperspectral indexes on day 2 of three light and temperature treatments for contrasting soil fertilities in three Miscanthus accessions.

Fig. S4. Diurnal response of hyperspectral indexes on day 7 of three light and temperature treatments for contrasting soil fertilities in three Miscanthus accessions.

Fig. S5. Diurnal response of the Carotenoid Reflectance Index (CRI; indicative of carotenoid content) on days 2 and 7 of three light and temperature treatments for contrasting soil fertilities in three Miscanthus accessions.

Fig. S6. Response of CRI in three Miscanthus genotypes grown in the field trial.

Table S1. Composition analysis of soil samples.

Table S2 Primer sequences used in RT–qPCR

Table S3. ANOVA for the first day of treatments.

Table S4. ANOVA for two diurnals on the second and seventh day of treatments.

Table S5. RT–qPCR cycle numbers.

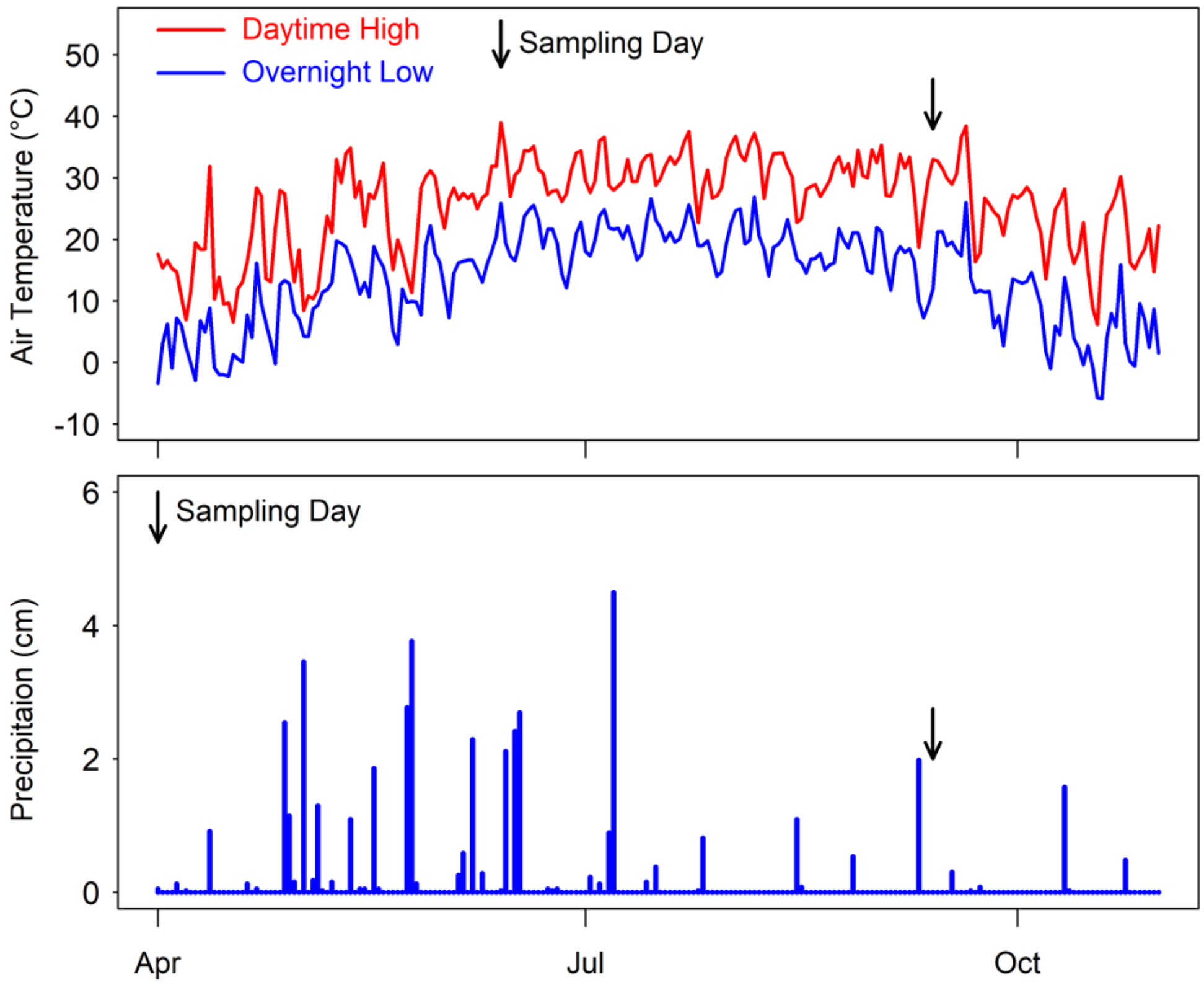

Fig. S1. Daytime high and overnight low temperatures (°C), along with daily precipitations (cm) collected for the field trial. The field experiment was at the University of Nebraska-Lincoln East Campus, Lincoln, NE (40.829 N, 96.657 W).

ALT TEXT: Panels present daytime high (top panel, top line) and overnight low temperatures (top panel bottom line) and daily precipitation (bottom panel) from April to November 2022 at the field location. Arrows indicate sampling date.

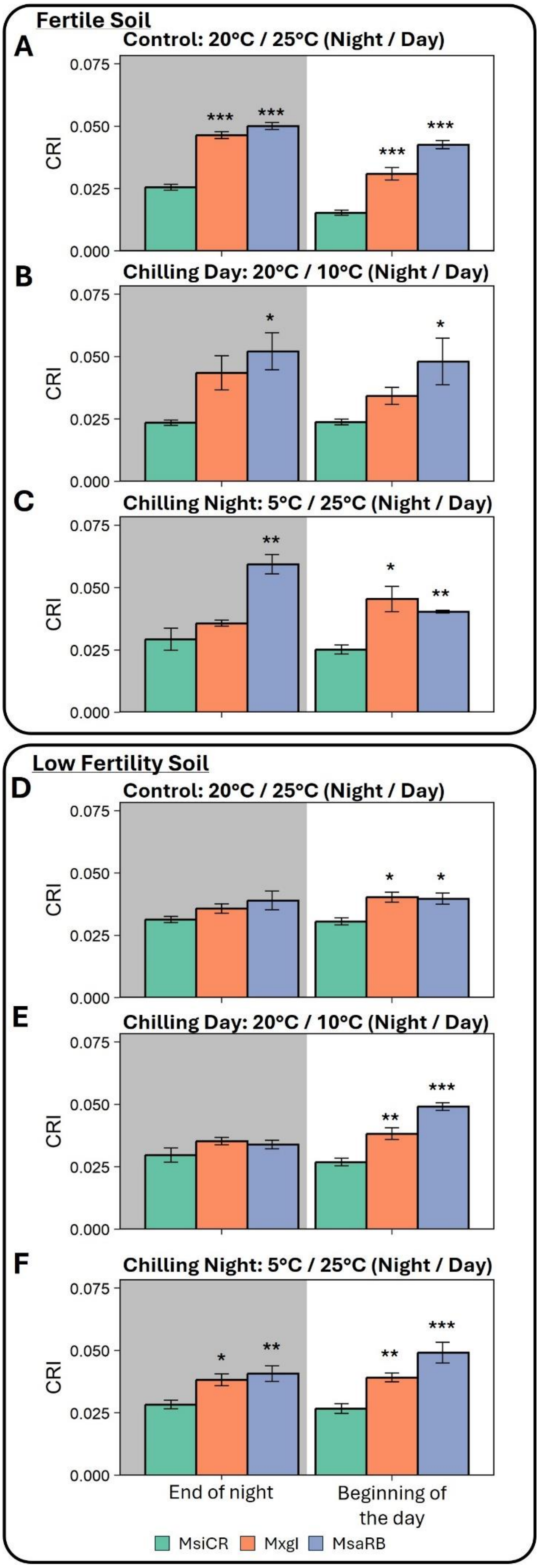

Fertile Soil
A
Control: 20°C / 25°C (Night / Day)
B
Chilling Day: 20°C / 10°C (Night / Day)
C
Chilling Night: 5°C / 25°C (Night / Day)
Low Fertility Soil
D
Control: 20°C / 25°C (Night / Day)
E
Chilling Day: 20°C / 10°C (Night / Day)
F
Chilling Night: 5°C / 25°C (Night / Day)
CRI
0.000
0.025
0.050
0.075
End of night
Beginning of the day
MsiCR
Mxgl
MsaRB

Fig. S2. Response of the Carotenoid Reflectance Index (CRI; positively correlated with carotenoid content) to soil fertility with the combination of light and temperature treatments in three *Miscanthus* accessions. Plants were grown in growth chambers under control conditions (20°C/25°C night/day) in (A-C) fertile or (D-F) low fertility soil for three weeks and then subjected to three different temperature and light combinations: control (20°C/25°C night/day), warm night with chilling day (20°C/10°C night/day) or chilling night with warm day (5°C/25°C night/day). CRI was measured on the youngest fully expanded leaf of *M. sinensis* (MsiCR; low chilling tolerance), *M.* × *giganteus* (MxgI; moderate chilling tolerance) and *M. sacchariflorus* (MsaRB; high chilling tolerance) at the end of first night (9 h of night) and beginning of the first day (15 min after the start of photoperiod). All data are means (± SE) with n = 4 biological replicates. Significant differences compared with MsiCR were determined using Dunnett's test: * $P \leq 0.05$; ** $P \leq 0.01$; *** $P \leq 0.001$.

ALT TEXT: Panels labelled A to F represent the sets of bar graphs with the shaded left side representing night conditions (end of the night), and the non-shaded right side representing day (the beginning of the following day) conditions. Each panel represents the Carotenoid Reflectance Index (CRI). Panels A to C show measurements for three *Miscanthus* accessions grown on fertile soil. Panels D to F show measurements for three *Miscanthus* accessions grown on low fertility soil. A and D show the measurements obtained in control conditions (20°C night and 25°C day), B and E show the measurements obtained in chilling day conditions (20°Cnight and 10°C day) and C and F show the measurements obtained in chilling night conditions (5°C night and 25°C day). In each set of bar graphs, the order of accessions (from left to right) is *M. sinensis* (MsiCR; low chilling tolerance), *M.* × *giganteus* (MxgI; moderate chilling tolerance) and *M. sacchariflorus* (MsaRB; high chilling tolerance).

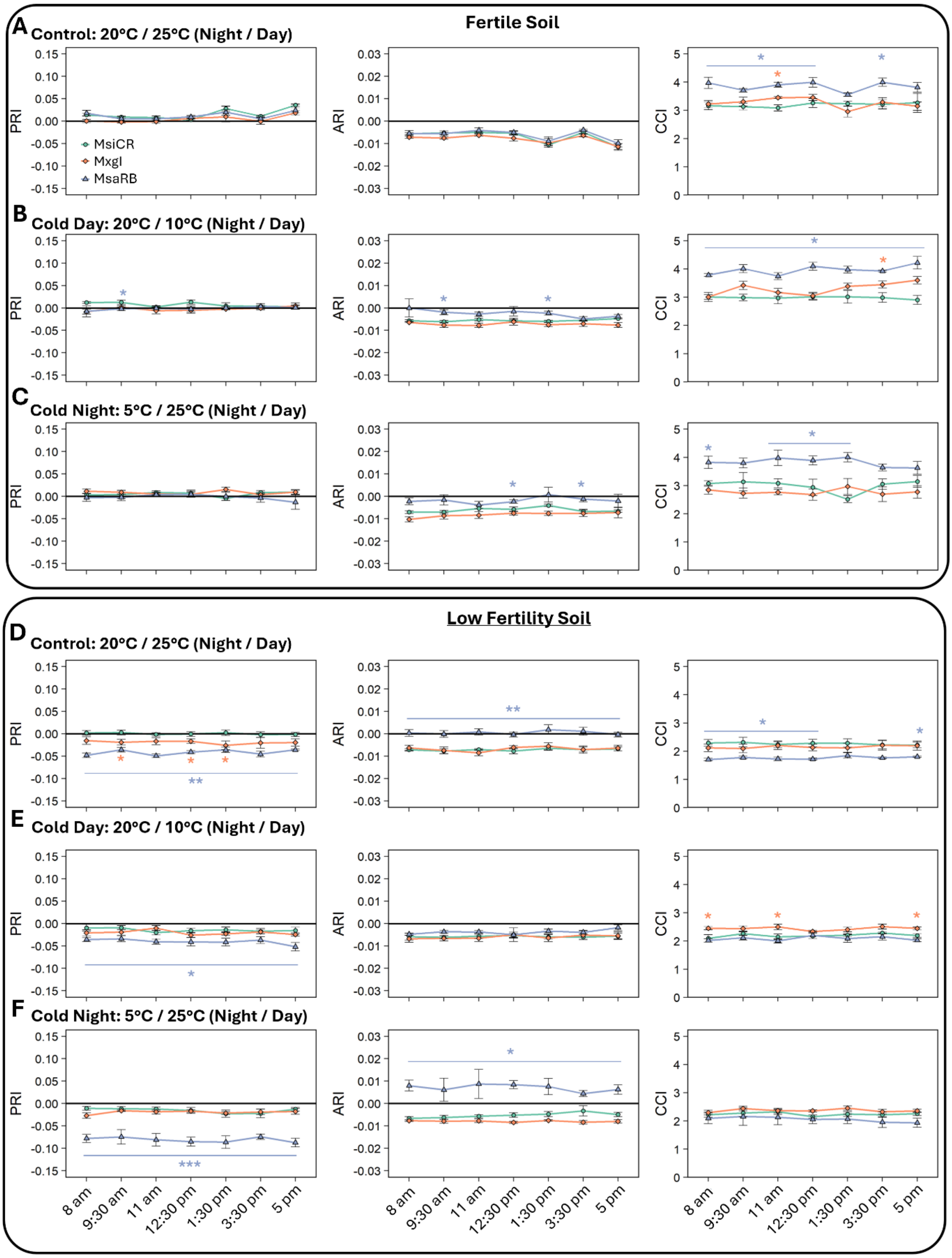

Fig. S3. Diurnal response of hyperspectral indexes on day 2 of three light and temperature treatments for contrasting soil fertilities in three *Miscanthus* accessions. Plants were grown in growth chambers under control conditions (20°C/25°C night/day) in (A-C) fertile or (D-F) low fertility soil for three weeks and then subjected to three different temperature and light combinations: control

(20°C/25°C night/day), warm night with chilling day (20°C/10°C night/day) or chilling night with warm day (5°C/25°C night/day). Hyperspectral indexes were measured on the youngest fully expanded leaf of *M. sinensis* (MsiCR; low chilling tolerance), *M.* × *giganteus* (MxgI; moderate chilling tolerance) and *M. sacchariflorus* (MsaRB; high chilling tolerance) on the 2nd day of treatment at seven timepoints: 1, 2.5, 4, 5.5, 6.5, 8.5 and 10 h following the start of light that corresponds to 8 am, 9.30 am, 11 am, 12.30 pm, 1.30 pm, 3.30 pm and 5 pm. Photochemical Reflectance Index (PRI; negatively correlated with de-epoxidation state of the xanthophylls), Anthocyanin Reflectance Index (ARI; positively correlated with anthocyanin content) and Chlorophyll Content Index (CCI; positively correlated with total chlorophyll content). All data are means (± SE) with n = 4 biological replicates. Significant differences compared with MsiCR were determined using Dunnett's test: * $P \leq 0.05$; ** $P \leq 0.01$; *** $P \leq 0.001$. The blue and orange line/asterisk above the curves represent significant difference between MsaRB (blue) or MxgI (orange) with MsiCR.

ALT TEXT:

Panels labelled A to F represent the sets of scatter graphs that show the hyperspectral index dynamics in leaves throughout the second day of treatment at 8 am, 9.30 am, 11 am, 12.30 pm, 1.30 pm, 3.30 pm and 5 pm, corresponding to 1, 2.5, 4, 5.5, 6.5, 8.5 and 10 h following the start of light, where the x-axis shows time and the y-axis shows hyperspectral index values. Individual symbols show mean measurements for the given time point and lines between symbols show the trend line. In each panel, three sets of graphs (from left to right) represent the Photochemical Reflectance Index (PRI), Anthocyanin Reflectance Index (ARI), and Chlorophyll Content Index (CCI). Panels A to C show measurements for three *Miscanthus* accessions grown on fertile soil. Panels D to F show measurements for three *Miscanthus* accessions grown on low fertility soil. A and D show the measurements obtained in control conditions (20°C night and 25°C day), B and E show the measurements obtained in chilling day conditions (20°Cnight and 10°C day) and C and F show the measurements obtained in chilling night conditions (5°C night and 25°C day).

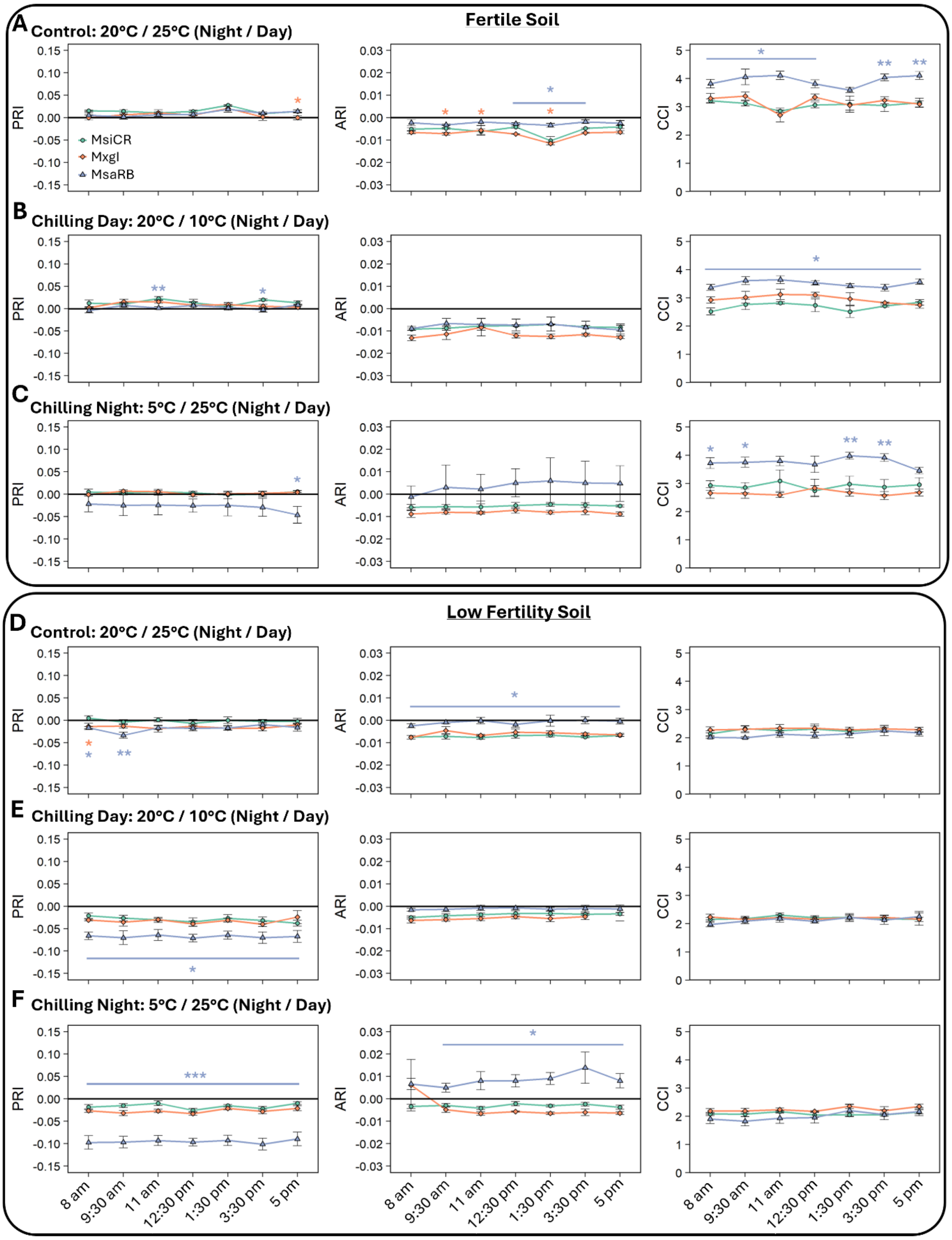

Fig. S4. Diurnal response of hyperspectral indexes on day 7 of three light and temperature treatments for contrasting soil fertilities in three *Miscanthus* accessions. Plants were grown in growth chambers under control conditions (20°C/25°C night/day) in (A-C) fertile or (D-F) low fertility soil for three weeks and then subjected to three different temperature and light combinations: control

(20°C/25°C night/day), warm night with chilling day (20°C/10°C night/day) or chilling night with warm day (5°C/25°C night/day). Hyperspectral indexes were measured on the youngest fully expanded leaf of *M. sinensis* (MsiCR; low chilling tolerance), *M.* × *giganteus* (MxgI; moderate chilling tolerance) and *M. sacchariflorus* (MsaRB; high chilling tolerance) on the 7th day of treatment at seven points: 1, 2.5, 4, 5.5, 6.5, 8.5 and 10 h following the start of light that corresponds to 8 am, 9.30 am, 11 am, 12.30 pm, 1.30 pm, 3.30 pm and 5 pm. Photochemical Reflectance Index (PRI; negatively correlated with de-epoxidation state of the xanthophylls), Anthocyanin Reflectance Index (ARI; positively correlated with anthocyanin content) and Chlorophyll Content Index (CCI; positively correlated with total chlorophyll content). All data are means (± SE) with n = 4 biological replicates. Significant differences compared with MsiCR were determined using Dunnett's test: * $P \leq 0.05$; ** $P \leq 0.01$; *** $P \leq 0.001$. The blue and orange line/asterisk above the curves represent significant difference between MsaRB (blue) or MxgI (orange) with MsiCR.

ALT TEXT:

Panels labelled A to F represent the sets of scatter graphs that show the hyperspectral index dynamics in leaves throughout the seventh day of treatment at 8 am, 9.30 am, 11 am, 12.30 pm, 1.30 pm, 3.30 pm and 5 pm, corresponding to 1, 2.5, 4, 5.5, 6.5, 8.5 and 10 h following the start of light, where the x-axis shows time and the y-axis shows hyperspectral index values. Individual symbols show mean measurements for the given time point and lines between symbols show the trend line. In each panel, three sets of graphs (from left to right) represent the Photochemical Reflectance Index (PRI), Anthocyanin Reflectance Index (ARI), and Chlorophyll Content Index (CCI). Panels A to C show measurements for three *Miscanthus* accessions grown on fertile soil. Panels D to F show measurements for three *Miscanthus* accessions grown on low fertility soil. A and D show the measurements obtained in control conditions (20°C night and 25°C day), B and E show the measurements obtained in chilling day conditions (20°Cnight and 10°C day) and C and F show the measurements obtained in chilling night conditions (5°C night and 25°C day).

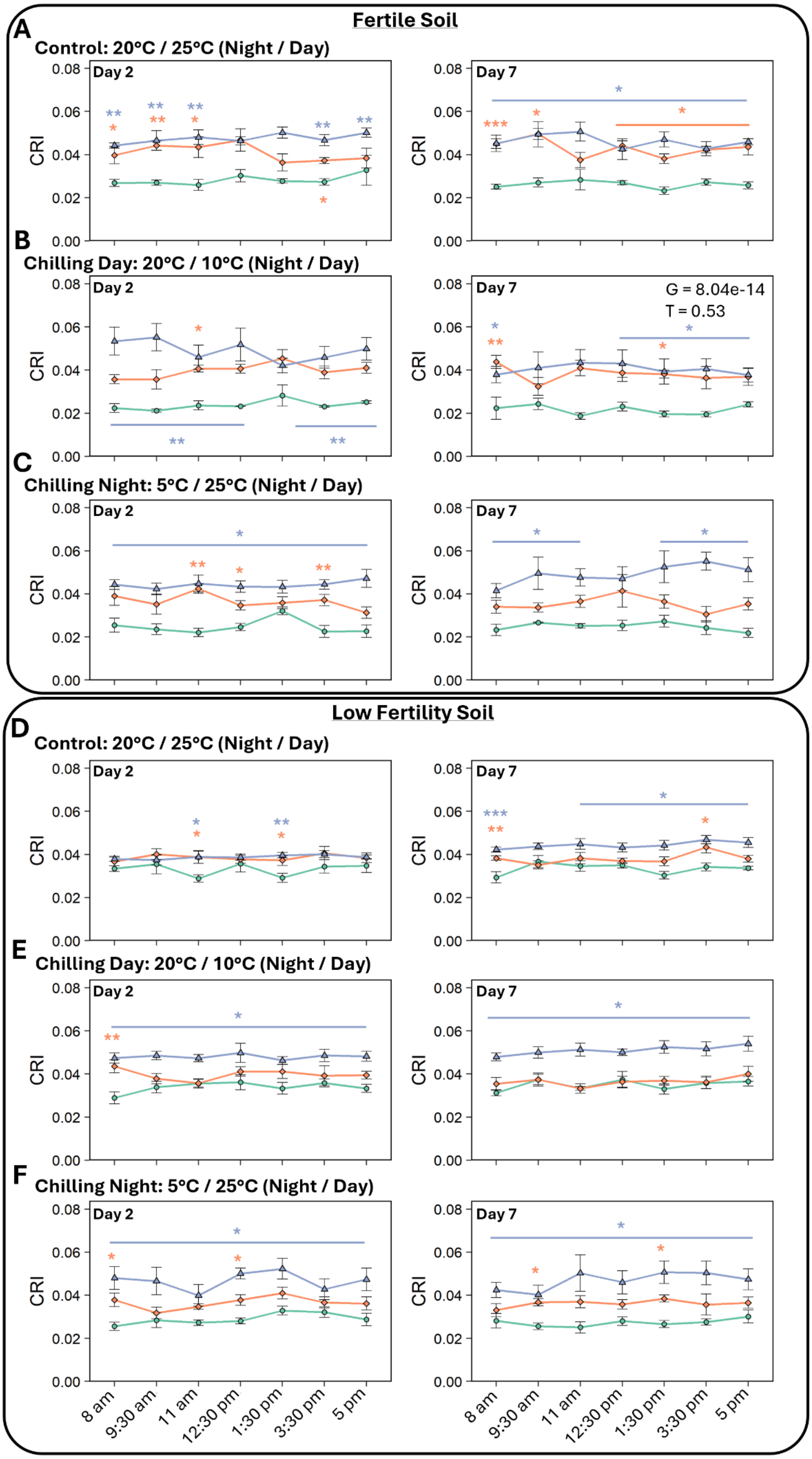

Fertile Soil
A
Control: 20°C / 25°C (Night / Day)
B
Chilling Day: 20°C / 10°C (Night / Day)
G = 8.04e-14
T = 0.53
C
Chilling Night: 5°C / 25°C (Night / Day)
Low Fertility Soil
D
Control: 20°C / 25°C (Night / Day)
E
Chilling Day: 20°C / 10°C (Night / Day)
F
Chilling Night: 5°C / 25°C (Night / Day)
Day 2
Day 7
CRI
8 am
9:30 am
11 am
12:30 pm
1:30 pm
3:30 pm
5 pm

Fig. S5. Diurnal response of the Carotenoid Reflectance Index (CRI; positively correlated with carotenoid content) on days 2 and 7 of three light and temperature treatments for contrasting soil fertilities in three *Miscanthus* accessions. Plants were grown in growth chambers under control conditions (20°C/25°C night/day) in (A-C) fertile or (D-F) low fertility soil for three weeks and then subjected to three different temperature and light combinations: control (20°C/25°C night/day), warm night with chilling day (20°C/10°C night/day) or chilling night with warm day (5°C/25°C night/day). CRI was measured on the youngest fully expanded leaf of *M. sinensis* (MsiCR; low chilling tolerance), *M.* × *giganteus* (MxgI; moderate chilling tolerance) and *M. sacchariflorus* (MsaRB; high chilling tolerance) on the 2nd and 7th day of treatment at seven points: 1, 2.5, 4, 5.5, 6.5, 8.5 and 10 h following the start of light that corresponds to 8 am, 9.30 am, 11 am, 12.30 pm, 1.30 pm, 3.30 pm and 5 pm. All data are means (± SE) with n = 4 biological replicates. Significant differences compared with MsiCR were determined using Dunnett's test: * $P \leq 0.05$; ** $P \leq 0.01$; *** $P \leq 0.001$. The blue and orange line/asterisk above the curves represent significant difference between MsaRB (blue) or MxgI (orange) with MsiCR.

ALT TEXT: Panels labelled A to F represent the sets of scatter graphs that show the hyperspectral index dynamics in leaves throughout the second (left column) and the seventh (right column) day of treatment at 8 am, 9.30 am, 11 am, 12.30 pm, 1.30 pm, 3.30 pm and 5 pm, corresponding to 1, 2.5, 4, 5.5, 6.5, 8.5 and 10 h following the start of light, where the x-axis shows time and the y-axis shows hyperspectral index values. Individual symbols show mean measurements for the given time point and lines between symbols show the trend line Each panel represents the Carotenoid Reflectance Index (CRI). Panels A to C show measurements for three *Miscanthus* accessions grown on fertile soil. Panels D to F show measurements for three *Miscanthus* accessions grown on low fertility soil. A and D show the measurements obtained in control conditions (20°C night and 25°C day), B and E show the measurements obtained in chilling day conditions (20°Cnight and 10°C day) and C and F show the measurements obtained in chilling night conditions (5°C night and 25°C day).

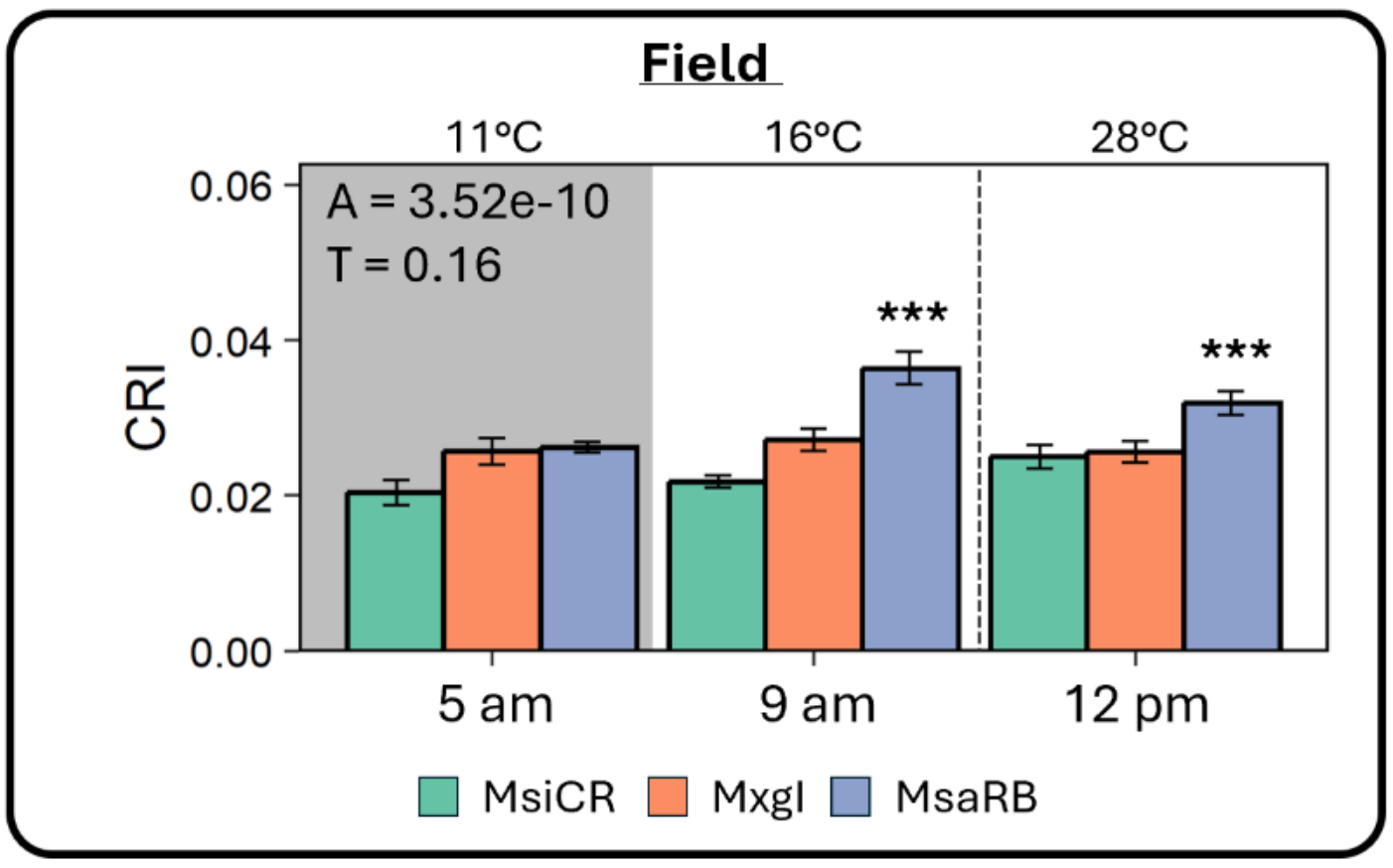

Fig. S6. Response of Carotenoid Reflectance Index (CRI; positively correlated with carotenoid content) in three *Miscanthus* genotypes grown in the field trial. Leaf reflectance was measured predawn (5 am), midmorning (9 am) and at noon (12 pm) on the youngest fully expanded leaf of *M. sinensis* (MsiCR; low chilling tolerance), *M.* × *giganteus* (MxgI; moderate chilling tolerance) and *M. sacchariflorus* (MsaRB; high chilling tolerance) following the naturally occurring chilling event. The field was located at the University of Nebraska-Lincoln East Campus, Lincoln, NE (40.829 N, 96.657 W). The field was rain-fed, not fertilized and temperature overnight low and day high were 10°C and 35°C for B panel (for full weather data see Fig. S1). All data are means (± SE) with n = 6 biological replicates. The *P*-values indicate the effect of accession (A) and time (T) in the ANOVA. Significant differences compared with MsiCR were determined using Dunnett's test: *** $P \leq 0.001$.

ALT TEXT: Sets of bar graphs plotting Carotenoid Reflectance Index (CRI) measured on three *Miscanthus* accessions grown in the field trial. In each bar graph set three time points are represented (from left to right): 5 am (11°C; shaded area), 9 am (16°C; non-shaded area) and 12 pm (28°C; non-shaded area). In each set of bar graphs, the order of accessions (from left to right) is *M. sinensis* (MsiCR; low chilling tolerance), *M.* × *giganteus* (MxgI; moderate chilling tolerance) and *M. sacchariflorus* (MsaRB; high chilling tolerance).

Table S1. Composition analysis of soil samples from the fertile soil and low fertile soil used for the growth chamber experiment, and from the field location. Values are mean (n=3) +/- standard deviation (SD).

Soil type	Soil pH 1:1	Soluble Salts 1:1 mmho/cm	Organic Matter LOI %	Nitrate-N KCl ppm	Nitrate Lbs/Acre	Phosphorus M3 ppm P	Potassium NH4OAc ppm K	Calcium NH4OAc ppm Ca	Magnesium NH4OAc ppm Mg	Sodium NH4OAc ppm Na	Sum of Cations me/100g
Field	7.2 ±0.2	0.5 ±0.2	3.8 ±0.2	7.8 ±3.9	13.7 ±9.8	96.0 ±12.7	611.3 ±29.0	2759.3 ±287.9	399.3 ±24.9	14.7 ±3.9	18.8 ±1.3
Growth Chamber Fertile Soil	6.0 ±0.1	1.4 ±0.2	58.9 ±1 3	54.5 ±0.7	98.0 ±1.0	33.0 ±3.0	73.0 ±7.0	784.0 ±62.0	101.0 ±7.0	13.5 ±0.5	8.7 ±0.3
Growth Chamber Low Fertility Soil	6.2 ±0.1	0.6 ±0.1	2.4 ±1.1	22.0 ±1.4	39.7 ±2.6	13.3 ±0.5	50.7 ±5.4	499.7 ±604	63.7 ±7.0	18.3 ±3.1	4.9 ±0.9

Soil type	H Saturation %	K Saturation %	Ca Saturation %	Mg Saturation %	Na Saturation %	Sulfate M-3 ppm S	Soil Texture	Clay %	Sand %	Silt %	Silt and Clay
Field	0.0 ±0	8.3 ±0.9	73.3 ±2.9	17.7 ±2.1	0.0 ±0	17.2 ±9.2	Clay Loam	34.3 ±0.9	22.7 ±2.5	43.0 ±1.6	77.3 ±2.5
Growth Chamber Fertile Soil	0.0 ±0	2.0 ±0.0	45.0 ±2.0	9.5 ±0.5	1.0 ±0	133.0 ±6.2	Sandy Loam	15.0 ±5.0	49.0 ±11.0	36.0 ±6.0	51.0 ±11.0
Growth Chamber Low Fertility Soil	0.0 ±0	2.7 ±0.5	53.0 ±12.4	11.7 ±2.6	1.3 ±0.5	45.9 ±6.9	Sandy	3.0 ±0.0	93.3 ±0.9	3.7 ±0.9	6.7 ±0.9

Table S2. Primer sequences used in RT–qPCR

Target	Sense	Sequence (5'-3')
ZEP	Forward	CCCAGTCACAAGGGTCATTAG
ZEP	Reverse	TCGACTACATGGCTTCCATTC
VDE	Forward	TGTACTGCAAGCGTCAGATATT
VDE	Reverse	AGAAGGGACTACCAGAACTAC
PsbS	Forward	CATCATCGGCGAGATCATCA
PsbS	Reverse	CGAAGAAGAAGAGGACGTTGA
DFR	Forward	CCATCCGGTCATCTTGACG
DFR	Reverse	CAAGCCGACGGTGGAAG
NYE	Forward	GTACACGCTGACGCACAG
NYE	Reverse	ATGAGGTCGAGCAGGAAGT
GluTR	Forward	CCAGTGGAGATGCGTGAAA
GluTR	Reverse	AAGAACAGCAGCCTCTTCAATA
EF	Forward	ACCCCTGCTTGATGCTCTT
EF	Reverse	AACCGGGACAGTTCCAATAC
UB	Forward	TCTACCCTCCACCTTGTCTT
UB	Reverse	CTCAACCTCCAGTGTGATTGT

Table S3. ANOVA results for data collected during the first day of treatment showing the effects of tested factors and their interactions on the measured variables (* P ≤ 0.001; ** P ≤ 0.01; * P ≤ 0.05; ns, not significant).**

Factors and interactions	PRI P_value	Significance	ARI P_value	Significance	CCI P_value	Significance	CRI P_value	Significance
accession	8.70E-36	***	5.06318E-54	***	0.001289025	**	3.82978E-59	***
light	3.99E-05	***	0.016705753	*	0.650206706	ns	0.360441296	ns
soil	6.31E-28	***	1.93337E-06	***	5.5034E-198	***	0.283589476	ns
temperature	1.80E-08	***	0.131762972	ns	0.166671904	ns	0.084756311	ns
accession:light	1.52E-03	**	0.58902016	ns	0.268068542	ns	0.257420748	ns
accession:soil	1.08E-10	***	1.53526E-06	***	2.42316E-39	***	2.93552E-07	***
light:soil	6.13E-02	ns	3.43941E-11	***	0.000230719	***	3.78822E-07	***
accession:temperature	6.36E-02	ns	7.06993E-05	***	0.006664675	**	0.261345112	ns
light:temperature	6.83E-01	ns	0.068301744	ns	1.63571E-06	***	0.051214099	ns
soil:temperature	3.04E-05	***	4.15447E-08	***	0.579216797	ns	0.205155492	ns
accession:light:soil	5.53E-01	ns	0.047063948	*	0.002602798	**	0.00023706	***
accession:light:temperature	1.66E-04	***	0.006534114	**	0.004666856	**	0.359854233	ns
accession:soil:temperature	1.35E-01	ns	0.690431122	ns	0.770794777	ns	0.099087067	ns
light:soil:temperature	6.41E-02	ns	0.001788191	**	0.154634823	ns	0.552878922	ns
accession:light:soil:temperature	3.99E-01	ns	0.184268222	ns	0.11114689	ns	0.000459984	***

Table S4. ANOVA results for two diurnals on the second and seventh day of treatments showing the effects of tested factors and their interactions on the measured variables during (* P ≤ 0.001; ** P ≤ 0.01; * P ≤ 0.05; ns, not significant).**

Factors and interactions	PRI P_value	Significance	ARI P_value	Significance	CCI P_value	Significance	CRI P_value	Significance
accession	6.51E-160	***	1.78E-229	***	1.29E-44	***	0	***
timepoint	0.438798222	ns	0.248694298	ns	0.649716712	ns	0.059416351	ns
soil	0	***	1.19E-143	***	0	***	2.87E-22	***
treatment	6.13E-93	***	2.08E-29	***	0.000167125	***	1.55E-06	***
day	2.38E-39	***	7.98E-21	***	6.49E-07	***	4.34E-05	***
accession:timepoint	0.889027069	ns	0.978850596	ns	0.909277457	ns	0.767907542	ns
accession:soil	2.69E-58	***	7.21E-31	***	3.10E-214	***	5.43E-18	***
timepoint:soil	0.409369481	ns	0.094587427	ns	0.217575627	ns	0.004031236	**
accession:treatment	1.00E-96	***	4.50E-57	***	3.59E-09	***	9.28E-24	***
timepoint:treatment	0.262673545	ns	0.066402192	ns	0.380838075	ns	0.000925792	***
soil:treatment	6.79E-22	***	1.49E-44	***	1.21E-22	***	4.42E-15	***
accession:day	0.032201804	*	0.645093517	ns	0.135794	ns	0.373669542	ns
timepoint:day	0.217096212	ns	0.714854709	ns	0.984298562	ns	0.324649731	ns
soil:day	2.15E-05	***	1.30E-09	***	3.07E-11	***	0.002722971	**
treatment:day	2.99E-55	***	1.42E-31	***	5.43E-10	***	2.51E-16	***
accession:timepoint:soil	0.989667813	ns	0.972049385	ns	0.946321849	ns	0.486817831	ns
accession:timepoint:treatment	0.856708294	ns	0.672779438	ns	0.868559595	ns	0.070818147	ns
accession:soil:treatment	8.20E-41	***	2.69E-34	***	1.80E-15	***	1.56E-08	***
timepoint:soil:treatment	0.376904962	ns	0.004986932	**	0.772706898	ns	0.138660434	ns
accession:timepoint:day	0.904915339	ns	0.910793169	ns	0.835876628	ns	0.022461833	*
accession:soil:day	0.82917166	ns	0.068674762	ns	0.171783938	ns	0.006029287	**
timepoint:soil:day	0.112614479	ns	0.219198771	ns	0.765023636	ns	0.906599429	ns
accession:treatment:day	1.20E-07	***	0.02759093	*	0.000158926	***	0.010269346	*

Continued from previous page

timepoint:treatment:day	0.92946972	ns	0.533872321	ns	0.479939039	ns	0.741056442	ns
soil:treatment:day	3.06E-16	***	7.98E-30	***	0.000396849	***	0.000678854	***
accession:timepoint:soil:treatment	0.999277097	ns	0.792332848	ns	0.972501901	ns	0.596444437	ns
accession:timepoint:soil:day	0.516229241	ns	0.999593113	ns	0.569072661	ns	0.501755822	ns
accession:timepoint:treatment:day	0.920995892	ns	0.987579499	ns	0.958815759	ns	0.135195485	ns
accession:soil:treatment:day	0.001280973	**	4.15E-05	***	0.166984644	ns	4.09E-06	***
timepoint:soil:treatment:day	0.87885736	ns	0.387831536	ns	0.639499184	ns	0.971542735	ns
accession:timepoint:soil:treatment:day	0.958491192	ns	0.850984921	ns	0.793769848	ns	0.978353689	ns

Table S5. RT–qPCR cycle numbers for MsiEF, MsiUB, MsiDFR, MsiNYE and MsiGluTR, MsiZEP, MsiVDE and MsiPsbS

Accession	Light	Temperature	Target	Biological replicate	Technical replicate	Cq
MsaRB	Dark	Warm	EF	1	1	23.63212
MsaRB	Dark	Warm	EF	1	2	23.24443
MsaRB	Dark	Warm	EF	2	1	25.98215
MsaRB	Dark	Warm	EF	2	2	25.94455
MsaRB	Dark	Warm	EF	3	1	23.79978
MsaRB	Dark	Warm	EF	3	2	23.47206
MsaRB	Dark	Warm	EF	4	1	22.84201
MsaRB	Dark	Warm	EF	4	2	22.93524
MsaRB	Dark	Warm	EF	5	1	26.60713
MsaRB	Dark	Warm	EF	5	2	26.46216
MsaRB	Dark	Warm	EF	6	1	24.30795
MsaRB	Dark	Warm	EF	6	2	24.18451
MsaRB	Dark	Warm	NYE	1	1	30.22596
MsaRB	Dark	Warm	NYE	1	2	30.16634
MsaRB	Dark	Warm	NYE	2	1	31.93044
MsaRB	Dark	Warm	NYE	2	2	32.23979
MsaRB	Dark	Warm	NYE	3	1	30.758
MsaRB	Dark	Warm	NYE	3	2	30.39962
MsaRB	Dark	Warm	NYE	4	1	31.44666
MsaRB	Dark	Warm	NYE	4	2	30.49805
MsaRB	Dark	Warm	NYE	5	1	32.22964
MsaRB	Dark	Warm	NYE	5	2	32.3584
MsaRB	Dark	Warm	NYE	6	1	30.32395
MsaRB	Dark	Warm	NYE	6	2	30.30107
MsaRB	Dark	Warm	UB	1	1	29.00852
MsaRB	Dark	Warm	UB	1	2	29.27609
MsaRB	Dark	Warm	UB	2	1	31.37563
MsaRB	Dark	Warm	UB	2	2	31.24644
MsaRB	Dark	Warm	UB	3	1	29.06268
MsaRB	Dark	Warm	UB	3	2	28.78767
MsaRB	Dark	Warm	UB	4	1	29.34596
MsaRB	Dark	Warm	UB	4	2	29.07617
MsaRB	Dark	Warm	UB	5	1	31.10953
MsaRB	Dark	Warm	UB	5	2	30.90411
MsaRB	Dark	Warm	UB	6	1	30.32547
MsaRB	Dark	Warm	UB	6	2	30.55443
MsaRB	Dark	Warm	GluTR	1	1	21.38608
MsaRB	Dark	Warm	GluTR	1	2	21.30302
MsaRB	Dark	Warm	GluTR	2	1	23.76279
MsaRB	Dark	Warm	GluTR	2	2	23.76787

Continued from previous page

MsaRB	Dark	Warm	GluTR	3	1	21.36358
MsaRB	Dark	Warm	GluTR	3	2	21.88091
MsaRB	Dark	Warm	GluTR	5	1	23.54688
MsaRB	Dark	Warm	GluTR	5	2	23.27811
MsaRB	Dark	Warm	DFR	1	1	27.95694
MsaRB	Dark	Warm	DFR	1	2	29.85607
MsaRB	Dark	Warm	DFR	4	1	28.63114
MsaRB	Dark	Warm	DFR	4	2	28.8745
MsaRB	Dark	Warm	DFR	5	1	30.22036
MsaRB	Dark	Warm	DFR	5	2	31.5154
MsiCR	Dark	Warm	EF	1	1	22.33597
MsiCR	Dark	Warm	EF	1	2	22.28724
MsiCR	Dark	Warm	EF	2	1	22.66274
MsiCR	Dark	Warm	EF	2	2	22.58293
MsiCR	Dark	Warm	EF	3	1	23.27627
MsiCR	Dark	Warm	EF	3	2	23.30272
MsiCR	Dark	Warm	EF	4	1	24.10216
MsiCR	Dark	Warm	EF	4	2	23.92326
MsiCR	Dark	Warm	EF	5	1	23.46514
MsiCR	Dark	Warm	EF	5	2	23.1859
MsiCR	Dark	Warm	GluTR	1	1	24.75293
MsiCR	Dark	Warm	GluTR	1	2	24.71589
MsiCR	Dark	Warm	GluTR	2	1	22.57891
MsiCR	Dark	Warm	GluTR	2	2	22.37258
MsiCR	Dark	Warm	GluTR	3	1	23.17945
MsiCR	Dark	Warm	GluTR	3	2	23.32403
MsiCR	Dark	Warm	GluTR	4	1	24.95162
MsiCR	Dark	Warm	GluTR	4	2	24.83645
MsiCR	Dark	Warm	GluTR	5	1	22.61925
MsiCR	Dark	Warm	GluTR	5	2	22.62951
MsiCR	Dark	Warm	NYE	1	1	31.33125
MsiCR	Dark	Warm	NYE	1	2	30.35998
MsiCR	Dark	Warm	NYE	2	1	30.76197
MsiCR	Dark	Warm	NYE	2	2	31.98698
MsiCR	Dark	Warm	NYE	3	1	31.94154
MsiCR	Dark	Warm	NYE	3	2	32.00429
MsiCR	Dark	Warm	NYE	4	1	30.86221
MsiCR	Dark	Warm	NYE	4	2	30.23443
MsiCR	Dark	Warm	NYE	5	1	31.18383
MsiCR	Dark	Warm	NYE	5	2	31.05717
MsiCR	Dark	Warm	UB	1	1	27.78041
MsiCR	Dark	Warm	UB	1	2	27.81338
MsiCR	Dark	Warm	UB	2	1	28.20867

Continued from previous page

MsiCR	Dark	Warm	UB	2	2	28.03494
MsiCR	Dark	Warm	UB	3	1	28.73142
MsiCR	Dark	Warm	UB	3	2	29.14134
MsiCR	Dark	Warm	UB	4	1	29.49013
MsiCR	Dark	Warm	UB	4	2	29.20553
MsiCR	Dark	Warm	UB	5	1	28.36683
MsiCR	Dark	Warm	UB	5	2	28.8154
MsiCR	Dark	Warm	DFR	1	1	25.96542
MsiCR	Dark	Warm	DFR	1	2	25.84243
MsiCR	Dark	Warm	DFR	3	1	25.79935
MsiCR	Dark	Warm	DFR	3	2	26.53412
MsiCR	Dark	Warm	DFR	4	1	25.81166
MsiCR	Dark	Warm	DFR	4	2	26.01544
MsaRB	Field	Field	DFR	1	1	30.78988
MsaRB	Field	Field	DFR	1	2	30.19227
MsaRB	Field	Field	EF	1	1	24.36623
MsaRB	Field	Field	EF	1	2	24.18647
MsaRB	Field	Field	GluTR	1	1	24.16448
MsaRB	Field	Field	GluTR	1	2	24.16338
MsaRB	Field	Field	NYE	1	1	27.4169
MsaRB	Field	Field	NYE	1	2	27.59439
MsaRB	Field	Field	PsbS	1	1	22.23341
MsaRB	Field	Field	PsbS	1	2	22.2071
MsaRB	Field	Field	Ub	1	1	30.10296
MsaRB	Field	Field	Ub	1	2	30.23028
MsaRB	Field	Field	VDE	1	1	25.65621
MsaRB	Field	Field	VDE	1	2	25.71889
MsaRB	Field	Field	ZEP	1	1	23.65482
MsaRB	Field	Field	ZEP	1	2	23.53845
MsaRB	Field	Field	DFR	2	1	33.02144
MsaRB	Field	Field	DFR	2	2	33.31469
MsaRB	Field	Field	EF	2	1	24.91254
MsaRB	Field	Field	EF	2	2	25.00559
MsaRB	Field	Field	GluTR	2	1	24.53075
MsaRB	Field	Field	GluTR	2	2	24.46177
MsaRB	Field	Field	NYE	2	1	29.80678
MsaRB	Field	Field	NYE	2	2	29.84592
MsaRB	Field	Field	PsbS	2	1	23.00533
MsaRB	Field	Field	PsbS	2	2	22.90164
MsaRB	Field	Field	Ub	2	1	30.23079
MsaRB	Field	Field	Ub	2	2	30.43069
MsaRB	Field	Field	VDE	2	1	26.20965
MsaRB	Field	Field	VDE	2	2	26.12725

Continued from previous page

MsaRB	Field	Field	ZEP	2	1	23.82873
MsaRB	Field	Field	ZEP	2	2	23.87117
MsaRB	Field	Field	DFR	3	1	30.07501
MsaRB	Field	Field	DFR	3	2	29.84813
MsaRB	Field	Field	EF	3	1	25.13345
MsaRB	Field	Field	EF	3	2	25.26117
MsaRB	Field	Field	GluTR	3	1	25.169
MsaRB	Field	Field	GluTR	3	2	25.2069
MsaRB	Field	Field	NYE	3	1	27.20392
MsaRB	Field	Field	NYE	3	2	27.63279
MsaRB	Field	Field	PsbS	3	1	22.34236
MsaRB	Field	Field	PsbS	3	2	22.41909
MsaRB	Field	Field	Ub	3	1	30.22846
MsaRB	Field	Field	Ub	3	2	30.45177
MsaRB	Field	Field	VDE	3	1	26.66655
MsaRB	Field	Field	VDE	3	2	27.06344
MsaRB	Field	Field	ZEP	3	1	24.47344
MsaRB	Field	Field	ZEP	3	2	24.64452
MsaRB	Field	Field	DFR	4	1	25.83857
MsaRB	Field	Field	DFR	4	2	25.90578
MsaRB	Field	Field	EF	4	1	24.53972
MsaRB	Field	Field	EF	4	2	24.43601
MsaRB	Field	Field	GluTR	4	1	24.04197
MsaRB	Field	Field	GluTR	4	2	24.07984
MsaRB	Field	Field	NYE	4	1	26.90152
MsaRB	Field	Field	NYE	4	2	27.00568
MsaRB	Field	Field	PsbS	4	1	21.72023
MsaRB	Field	Field	PsbS	4	2	21.72705
MsaRB	Field	Field	Ub	4	1	29.52127
MsaRB	Field	Field	Ub	4	2	29.33269
MsaRB	Field	Field	VDE	4	1	25.72116
MsaRB	Field	Field	VDE	4	2	25.74867
MsaRB	Field	Field	ZEP	4	1	23.19519
MsaRB	Field	Field	ZEP	4	2	23.27811
MsaRB	Field	Field	DFR	5	1	36.17203
MsaRB	Field	Field	EF	5	1	26.61618
MsaRB	Field	Field	EF	5	2	26.40536
MsaRB	Field	Field	GluTR	5	1	26.82642
MsaRB	Field	Field	GluTR	5	2	26.7348
MsaRB	Field	Field	NYE	5	1	30.70758
MsaRB	Field	Field	NYE	5	2	30.64789
MsaRB	Field	Field	PsbS	5	1	25.04092
MsaRB	Field	Field	PsbS	5	2	24.99112

Continued from previous page

MsaRB	Field	Field	Ub	5	1	33.2098
MsaRB	Field	Field	Ub	5	2	33.28752
MsaRB	Field	Field	VDE	5	1	28.53853
MsaRB	Field	Field	VDE	5	2	28.5846
MsaRB	Field	Field	ZEP	5	1	26.01763
MsaRB	Field	Field	ZEP	5	2	26.02176
MsiCR	Field	Field	DFR	1	1	29.64823
MsiCR	Field	Field	DFR	1	2	30.19521
MsiCR	Field	Field	EF	1	1	23.34335
MsiCR	Field	Field	EF	1	2	23.41758
MsiCR	Field	Field	GluTR	1	1	24.94461
MsiCR	Field	Field	GluTR	1	2	24.98101
MsiCR	Field	Field	NYE	1	1	28.5042
MsiCR	Field	Field	NYE	1	2	28.39664
MsiCR	Field	Field	PsbS	1	1	22.05675
MsiCR	Field	Field	PsbS	1	2	22.12805
MsiCR	Field	Field	Ub	1	1	29.74251
MsiCR	Field	Field	Ub	1	2	29.87188
MsiCR	Field	Field	VDE	1	1	25.89681
MsiCR	Field	Field	VDE	1	2	26.11291
MsiCR	Field	Field	ZEP	1	1	23.12313
MsiCR	Field	Field	ZEP	1	2	23.22393
MsiCR	Field	Field	DFR	2	1	35.22444
MsiCR	Field	Field	DFR	2	2	35.55181
MsiCR	Field	Field	EF	2	1	23.9625
MsiCR	Field	Field	EF	2	2	23.67607
MsiCR	Field	Field	GluTR	2	1	24.63034
MsiCR	Field	Field	GluTR	2	2	24.66961
MsiCR	Field	Field	NYE	2	1	33.21179
MsiCR	Field	Field	NYE	2	2	32.67461
MsiCR	Field	Field	PsbS	2	1	22.96468
MsiCR	Field	Field	PsbS	2	2	23.01106
MsiCR	Field	Field	Ub	2	1	29.40886
MsiCR	Field	Field	Ub	2	2	29.43267
MsiCR	Field	Field	VDE	2	1	26.17859
MsiCR	Field	Field	VDE	2	2	26.04951
MsiCR	Field	Field	ZEP	2	1	23.89883
MsiCR	Field	Field	ZEP	2	2	23.85004
MsiCR	Field	Field	DFR	3	1	35.86298
MsiCR	Field	Field	DFR	3	2	35.9594
MsiCR	Field	Field	EF	3	1	24.39789
MsiCR	Field	Field	EF	3	2	24.70337
MsiCR	Field	Field	GluTR	3	1	25.16271

Continued from previous page

MsiCR	Field	Field	GluTR	3	2	25.17881
MsiCR	Field	Field	NYE	3	1	32.57434
MsiCR	Field	Field	NYE	3	2	32.56682
MsiCR	Field	Field	PsbS	3	1	22.83553
MsiCR	Field	Field	PsbS	3	2	23.09152
MsiCR	Field	Field	Ub	3	1	30.55634
MsiCR	Field	Field	Ub	3	2	30.30295
MsiCR	Field	Field	VDE	3	1	27.00446
MsiCR	Field	Field	VDE	3	2	26.93983
MsiCR	Field	Field	ZEP	3	1	23.31496
MsiCR	Field	Field	ZEP	3	2	24.89423
MsiCR	Field	Field	DFR	4	1	33.16548
MsiCR	Field	Field	DFR	4	2	33.06937
MsiCR	Field	Field	EF	4	1	26.04278
MsiCR	Field	Field	EF	4	2	26.07494
MsiCR	Field	Field	GluTR	4	1	26.2485
MsiCR	Field	Field	GluTR	4	2	26.26998
MsiCR	Field	Field	NYE	4	1	34.82376
MsiCR	Field	Field	NYE	4	2	35.31775
MsiCR	Field	Field	PsbS	4	1	24.31832
MsiCR	Field	Field	PsbS	4	2	24.2429
MsiCR	Field	Field	Ub	4	1	31.31112
MsiCR	Field	Field	Ub	4	2	30.99368
MsiCR	Field	Field	VDE	4	1	28.11521
MsiCR	Field	Field	VDE	4	2	28.19354
MsiCR	Field	Field	ZEP	4	1	25.54277
MsiCR	Field	Field	ZEP	4	2	25.52643
MsiCR	Field	Field	DFR	6	1	39.19259
MsiCR	Field	Field	EF	6	1	26.99914
MsiCR	Field	Field	EF	6	2	26.91347
MsiCR	Field	Field	GluTR	6	1	27.26665
MsiCR	Field	Field	GluTR	6	2	27.3509
MsiCR	Field	Field	NYE	6	1	36.76704
MsiCR	Field	Field	NYE	6	2	37.03413
MsiCR	Field	Field	PsbS	6	1	25.09445
MsiCR	Field	Field	PsbS	6	2	25.09069
MsiCR	Field	Field	Ub	6	1	33.06552
MsiCR	Field	Field	Ub	6	2	33.11416
MsiCR	Field	Field	VDE	6	1	29.29748
MsiCR	Field	Field	VDE	6	2	29.30692
MsiCR	Field	Field	ZEP	6	1	26.31448
MsiCR	Field	Field	ZEP	6	2	26.33837
MsiCR	Field	Field	DFR	5	1	33.13282

Continued from previous page

MsiCR	Field	Field	DFR	5	2	34.3138
MsiCR	Field	Field	EF	5	1	25.68079
MsiCR	Field	Field	EF	5	2	25.67579
MsiCR	Field	Field	GluTR	5	1	25.68621
MsiCR	Field	Field	GluTR	5	2	25.73033
MsiCR	Field	Field	NYE	5	1	32.48486
MsiCR	Field	Field	NYE	5	2	32.95778
MsiCR	Field	Field	PsbS	5	1	23.46546
MsiCR	Field	Field	PsbS	5	2	23.61738
MsiCR	Field	Field	Ub	5	1	31.42401
MsiCR	Field	Field	Ub	5	2	31.57034
MsiCR	Field	Field	VDE	5	1	28.0694
MsiCR	Field	Field	VDE	5	2	28.01244
MsiCR	Field	Field	ZEP	5	1	25.21874
MsiCR	Field	Field	ZEP	5	2	25.06765
